# Chemical Constituents from the Aerial Parts of *Cyrtopodium paniculatum*

**DOI:** 10.3390/molecules21101418

**Published:** 2016-10-24

**Authors:** Florence Auberon, Opeyemi Joshua Olatunji, Gaëtan Herbette, Diamondra Raminoson, Cyril Antheaume, Beatriz Soengas, Frédéric Bonté, Annelise Lobstein

**Affiliations:** 1 Laboratory of Pharmacognosy and Bioactive Natural Products, Faculty of Pharmacy, Strasbourg University, Illkirch-Graffenstaden 67400, France; pere@fastermail.com (O.J.O.); harimisad.raminoson@gmail.com (D.R.); lobstein@unistra.fr (A.L.); 2 Spectropôle, FR1739, Aix-Marseille University, Campus de St Jérôme-Service 511, Marseille 13397, France; gaetan.herbette@univ-amu.fr; 3 LIVE group, New-Caledonia University, BP R4, Noumea Cedex 98851, New-Caledonia; cyril.antheaume@univ-nc.nc; 4 LVMH Recherche, 185 avenue de Verdun, St Jean de Braye 45800, France; bsoengas@research.lvmh-pc.com (B.S.); fredericbonte@research.lvmh-pc.com (F.B.)

**Keywords:** *Cyrtopodium paniculatum*, Orchidaceae, stilbenoids, phenanthrenes derivatives, biphenanthrenes, dihydrophenanthrenofurans

## Abstract

We report the first phytochemical study of the neotropical orchid *Cyrtopodium paniculatum*. Eight new compounds, including one phenanthrene **1**, one 9,10-dihydro-phenanthrene **2**, one hydroxybenzylphenanthrene **3**, two biphenanthrenes **4**–**5**, and three 9,10 dihydrophenanthrofurans **6**–**8**, together with 28 known phenolic compounds, mostly stilbenoids, were isolated from the CH_2_Cl_2_ extract of its leaves and pseudobulbs. The structures of the new compounds were established on the basis of extensive spectroscopic methods.

## 1. Introduction

The family Orchidaceae comprises 820 genera with almost 35,000 described species and can be regarded as the largest family of flowering plants. The phytochemical and biological investigations of this family has been mainly conducted in relation with their traditional uses and focused on a large number of Asian orchids from the genus *Arundina*, *Bletilla*, *Dendrobium*, *Gastrodia* and *Pleione* at the expense of the New World orchids In fact, the first report on a phytochemical study of South American orchids was in the late 1990s and was mainly focused on species in the genus *Scaphyglottis* [1,2,3], *Maxillaria* [4,5,6,7] and *Cyrtopodium* [8,9,10].

This genus *Cyrtopodium* includes 47 endemic species, distributed from Southern Florida to Central America, and they are terrestrial or epiphytic orchids, well recognized by their ovoid to fusiform pseudobulbs as well as their showy flowers [11,12,13,14]. To the best of our knowledge, only three *Cyrtopodium* species have been explored: *C. cardiochilum* Lindl. and *C. andersonnii* R.Br. for their polysaccharidic content, which displays anti-inflammatory and gastroprotective activities [8,10], and *C. macrobulbon* (La Llave & Lex.) G.A. Romero & Carnevali, a species traditionally used for treating urinary infections and whose stilbenoids are considered as its bioactive constituents [9]. *C. paniculatum* (Ruiz & Pav.) Garay has not yet received any attention since no report on its medicinal uses or chemical composition has been found in the literature. This provides a substantial basis for a detailed phytochemical investigation of this unexplored species. Therefore, in our continuing search for bioactive secondary metabolites from orchids [15,16,17], we carried out an investigation on the CH_2_Cl_2_ extracts from the leaves and pseudobulbs of *C. paniculatum*. This investigation led to the isolation of eight new compounds **1**–**8**, together with 28 known compounds. In this paper, we describe the structure elucidation of these new compounds, which was performed by means of NMR, HRESIMS data and CD spectrum determination.

## 2. Results and Discussion

### Structure Elucidation

**Figure 1:**
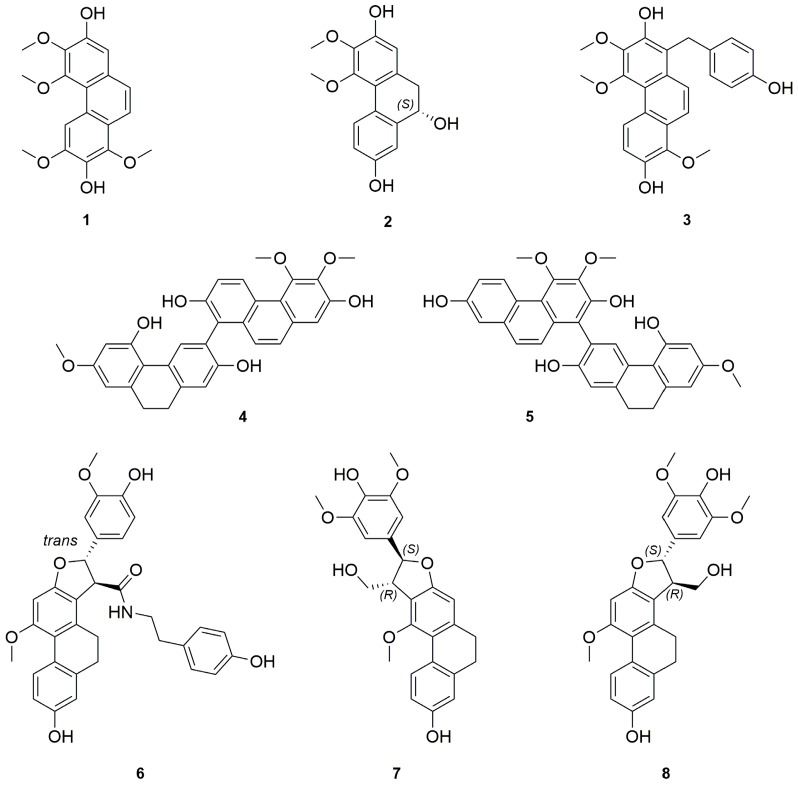
Chemical structures of compounds **1**–**8** from *C. paniculatum* pseudobulbs.

The pseudobulbs and the leaves from *C. paniculatum* were studied separately. The freshly cut pseudobulbs (7.5 kg) were ground and macerated in water to get rid of their sugar content, and then in ethanol to afford a crude extract (60 g). The alcoholic crude extract was suspended in water and sequentially extracted with cyclohexane, CH_2_Cl_2_ and *n*-BuOH. The CH_2_Cl_2_ extract was concentrated to dryness and subjected to silica gel column chromatography, Sephadex LH-20 and preparative thin layer chromatography (PTLC) to afford eight new compounds **1**–**8** (Figure 1) together with 28 previously described compounds **9**–**35** which were identified as *para*-ethoxybenzyl alcohol (**9**) [18,19], 3,4,6-trimethoxyl-9,10-dihydrophenanthrene-2,7-diol (**10**) [20], confusarin (**11**) [21], erianthridin (**12**) [22], *para*-hydroxybenzaldehyde (**13**) [18], nudol (**14**) [23], cephathrene-B (**15**) [24], gigantol (**16**) [25], batatasin III (**17**) [26], ephemeranthoquinone (**18**) [27], coelonin (**19**) [28], 2,4-dimethoxy-phenanthrene-3,7-diol (**20**) [29], lusianthridin (**21**) [30], densiflorol B (**22**) [31], denthyrsinin (**23**) [32], bleformins A (**24**) and B (**25**) [33], chrysoeriol (**26**) [34,35], vanillic alcool (**27**) [36], gastrodigenin (**28**) [37,38], 1-(4-hydroxybenzyl)-4-methoxyl-9,10-dihydrophenanthrene-2,7-diol (**29**) [39], shancidin (**30**) [40], blestriarenes A (**32**) and B (**31**) [41], velutin (**33**) [42], (+)-syringaresinol (**34**) [43] and (+)-balanophonin (**35**) [44], respectively, by comparison of their UV, MS, and NMR data with literature data.

In parallel, the leaves (70 g) were ground, lyophilized and successively extracted by maceration with cyclohexane, CH_2_Cl_2_ and *n*-BuOH. The CH_2_Cl_2_ extract (1.23 g) was fractionated using vacuum liquid chromatography (VLC) and semi-preparative RP-HPLC to afford gastrodigenin (**28**), coelonin (**19**), ephemeranthoquinone (**18**), lusianthridin (**21**), 1-(4-hydroxybenzyl)-4-methoxyl-9,10-dihydrophenanthrene-2,7-diol (**29**) and *trans*-feruloyltyramine (**36**) [45].

Compound **1** was obtained as a brown amorphous powder and showed a [M + H]^+^ peak at *m*/*z* 331.1188 (calcd. for C_18_H_19_O_6_ 331.1176) in the HRESIMS, indicating a molecular formula of C_18_H_18_O_6_ and suggesting the existence of ten degrees of unsaturation. The UV maximal absorptions at 216, 261, 281, 312 and 344 nm indicated the presence of a phenanthrene derivative [46,47,48]. The IR spectrum exhibited a broad peak at 3370 cm^−1^, characteristic of hydroxyl groups, and 1616, 1576, 953 and 860 cm^−1^, characteristic of aromatic rings. Comparison of the HRMS and the ^1^H-NMR data of **1** to those of cephathrene-B (**15**) [16] indicated substantial similarities between the two compounds, thus suggesting that compound **1** is structurally related to cephathrene-B. Extensive analysis of the 1D NMR of **1** (Table 1) indicated the presence of two mutually *ortho*-coupled aromatic protons at δ_H_ 7.88 (1H, d, *J* = 8.9 Hz, H-10) and 7.50 (1H, d, *J* = 8.9 Hz, H-9), thus suggesting the presence of a tetra-substituted aromatic ring. Additional aromatic proton signals at δ_H_ 8.90 (1H, s, H-4) and 7.17 (1H, s, H-8) afforded two penta-substituted aromatic rings. Two hydroxyl signals at δ_H_ 7.91 (1H, s, 2-OH) and 8.39 (1H, s, 7-OH) were observed. Four methoxyl groups were presumed based on the ^1^H-NMR resonances at δ_H_ 3.99 (3H, s, 1-OCH_3_), 4.06 (3H, s, 3-OCH_3_), 4.03 (3H, s, 5-OCH_3_) and 4.02 (3H, s, 6-OCH_3_) and was supported by the corresponding ^13^C-NMR signals at δ_C_ 61.1, 56.3, 60.5 and 61.4, respectively.

HMBC correlations from 1-OCH_3_ to C-1, 3-OCH_3_ to C-3, 5-OCH*_3_* to C-5 and 6-OCH_3_ to C-6 confirmed the position of the methoxyls assigned to C-1, C-3, C-5 and C-6 respectively. In the same way, the locations of the hydroxyl signals at δ_H_ 7.91 (1H, s, 2-OH) and 8.39 (1H, s, 7-OH) were positioned on C-2 and C-7 respectively, due to HMBC correlations from 2-OH to C-1, C-2 and C-3 and 7-OH with C-6, C-7, and C-8. Furthermore, NOESY correlations between 2-OH and 1/3-OCH_3_; 7-OH with 6-OCH_3_ and H-8 confirmed the positions of the hydroxyl and methoxyl substituents. Additional HMBC correlations from H-4 to C-2, C-3, C-4b, and C-10a; H-8 with C-4b, C-5, C-6, C-7 and C-9; from H-9 to C-4b, C-8 and C-10a and H-10 to C-1, C-4a and C-8a established the link between the three aromatic moieties. Therefore, **1** was assigned as 1,3,5,6-tetramethoxylphenanthrene-2,7-diol and named cyrtopodin.

Compound **2** was obtained as a white amorphous powder. Its molecular formula was determined as C_16_H_16_O_5_ as deduced from HRESIMS at *m*/*z* 311.0884 [M + Na]^+^; (calcd. for C_16_H_16_NaO_5_ 311.8900), suggesting the presence of nine degrees of unsaturation. The UV spectrum showed absorption maxima at 220, 262 and 282 nm suggesting a 9,10 dihydrophenanthrene moiety [49,50]. The IR spectrum showed a broad absorption at 3227 cm^−1^, indicating the presence of hydroxyl groups and at 1611, 1584, 999, 946, 870 and 828 cm^−1^, characteristic of aromatic rings. The ^1^H-NMR spectra (Table 1) showed a close resemblance to those of erianthridin (**12**) [22], except for the presence of an additional hydroxyl at position 9. This was supported by signals of the ^1^H and ^13^C resonance belonging to the methylene protons at δ_H_ 2.70 (1H, dd, *J* = 14.3, 10.6 Hz, H-10 ax) and 2.83 (1H, dd, *J* = 14.3, 4.6 Hz, H-10 eq), a deshielded methine resonance at δ_H_ 4.63 (1H, dd, *J* = 10.6, 4.6 Hz, H-9 ax), and deshielded carbon resonance at δ_C_ 68.9 (C-9). The absolute configuration of compound 2 was obtained with the help of circular dichroism (CD). The CD spectrum of **2** showed a negative Cotton effect at 236 nm and a positive Cotton effect at 281 nm, suggesting a 9*S* configuration for compound 2 which agrees with previous reports [51,52]. Therefore, **2** was identified as (+)-(*S*)-3,4-dimethoxyl-9,10-dihydrophenanthrene-2,7,9-triol, and named (*S*)-9-hydroxyerianthridin.

Compound **3** was obtained as a yellow amorphous powder. It showed a [M + H]^+^ signal at *m*/*z* 407.1508 (calcd. for C_24_H_23_O_6_ 407.1890) in the HRESIMS, indicating a molecular formula of C_24_H_22_O_6_ and fourteen degrees of unsaturation. Its UV spectrum showed maximal absorptions at 212, 268, 289, 297, 301 and 314 nm suggesting a phenanthrene moiety. The 1D-NMR data indicated that the structure of **3** comprised a confusarin (**11**) [21] and gastrodigenin (**28**) [38] subunits. This hypothesis was supported by the 1D and 2D-NMR spectral data (Table 1). In the ^1^H-NMR spectrum, six aromatic protons signals at δ_H_ 9.21 (1H, d, *J* = 9.4 Hz, H-5), 7.24 (1H, d, *J* = 9.4 Hz, H-6), 7.87 (1H, d, *J* = 9.5 Hz, H-9), 7.90 (1H, d, *J* = 9.5 Hz, H-10) and three methoxyl proton signals at δ_H_ 4.06 (3H, s, 3-OCH_3_), 3.95 (3H, s, 4-OCH_3_) and 3.90 (3H, s, 8-OCH_3_) were observed, which corresponds to the resonances attributable to the confusarin moiety.

**Figure 2:**
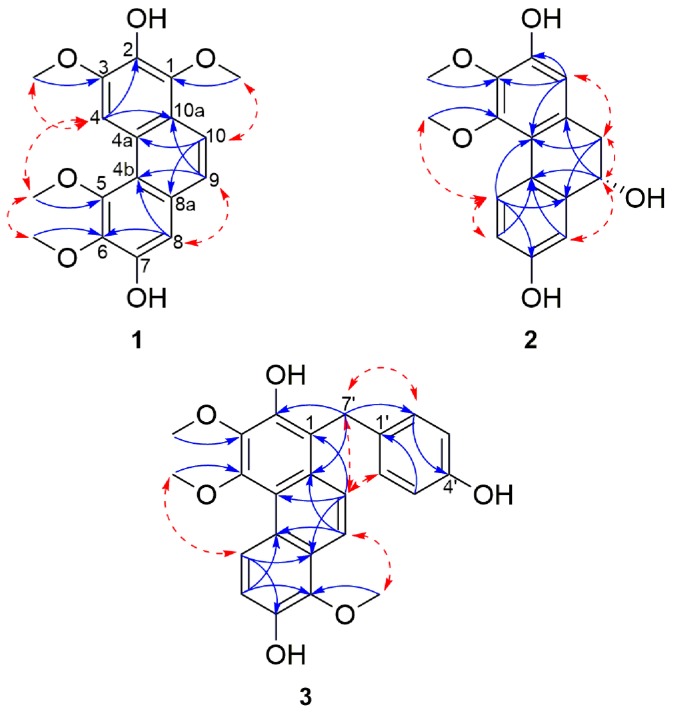
Selected NOESY (red dashed arrows) and HMBC (blue arrows) correlations of compounds **1**–**3**.

Additional aromatic signals of two equivalent set of mutually coupling protons at δ_H_ 7.08 (2H, d, *J* = 8.6 Hz, H-2′/6′) and 6.68 (2H, d, *J* = 8.6 Hz, H-3′/5′) indicated the presence of a di-substituted aromatic ring which was attributed to the resonance belonging to the gastrodigenin moiety. The linkage between the confusarin and gastrodigenin subunits took place via a methylene bridge at δ_H_ 4.41 (s, H-7′) and was confirmed by the HMBC correlations from H-7′ to C-2, C-10a, C-2′/6′ (Figure 2). Thus, compound **3** was identified as 1-(4′-hydroxybenzyl)-3,4,8-trimethoxylphenanthrene-2,7-diol and named gastrodiconfusarin.

**Figure 3:**
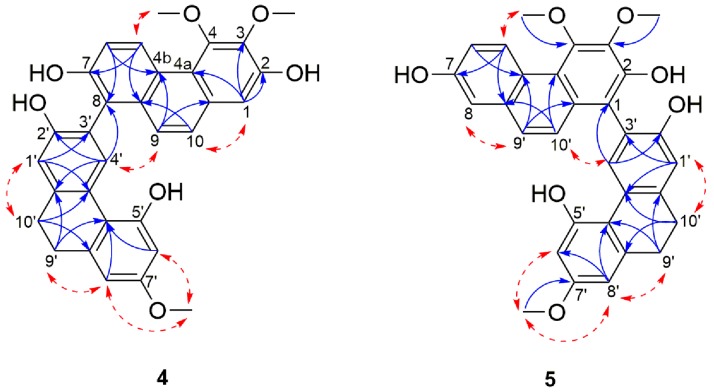
Selected NOESY (red dashed arrows) and HMBC (blue arrows) correlations of compounds **4**–**5**.

Compound **4** was obtained as a pale yellow amorphous powder. The HRESIMS showed a [M + H]^+^ molecular ion peak at *m*/*z* 511.1742 indicating a molecular formula of C_31_H_26_O_7_ (calcd. C_31_H_27_O_7_ for 511.1751) which is consistent with nineteen degrees of unsaturation. Its UV spectrum showed maximal absorptions at 213, 263, 296, 310, 349 and 367 nm. The IR spectrum showed typical absorption bands of a hydroxyl group at 3245 cm^−1^ and of aromatic rings at 1608, 1585, 948, 865 and 830 cm^−1^. In 1D-NMR spectrum (Table 2), the presence of two deshielded aromatic proton signals at δ_H_ 9.42 (1H, d, *J* = 9.2 Hz, H-5) and δ_H_ 8.28 (1H, s, H-4′) suggested that **4** was a dimeric molecule formed from the fusion of a phenanthrene unit [23,46,53] and a 9,10-dihydrophenanthrene subunit [28,54]. The ^1^H spectrum showed resonances for a pair of *ortho*-coupled aromatic protons at δ_H_ 9.42 (1H, d, *J =* 9.2 Hz, H-5) and 7.31 (1H, d, *J =* 9.2 Hz, H-6), a broad singlet integrated for two aromatic protons at δ_H_ 7.43 (2H, s, H-9, H-10), two meta-coupled aromatic protons at δ_H_ 6.39 (1H, d, *J* = 2.6 Hz, H-6′) and 6.42 (d, *J =* 2.6 Hz, H-8′) and three isolated aromatic protons at δ_H_ 7.11 (1H, s, H-1), 6.92 (1H, s, H-1′) and 8.28 (1H, s, H-4′). Additional signals belonging to three methoxyl groups at δ_H_ 4.01 (3H, s, 3-OCH_3_), 4.00 (3H, s, 4-OCH_3_), 3.73 (3H, s, 7′-OCH_3_) and two methylene groups emerging as a broad singlet at δ_H_ 2.80 (4H, s, H-9′ and H-10′), were also observed. The position of the hydroxyl and methoxyl functions and the linkage between the two subunits were ascertained based on the comprehensive analysis of ^13^C, HMBC and NOESY spectra (Figure 3). HMBC correlations from H-1 and 3-OCH_3_ to C-3, as well as HMBC cross peak from 4-OCH_3_ to C-4 and NOESY cross peak between 4-OCH_3_ and H-5 confirmed the location of the two methoxyl group on position C-3 and C-4 of the phenanthrene unit.

The positioning of the substituents on the 9,10-dihydrophenanthrene unit was deduced based on a HMBC cross peak from H-4′ to C-2′, as well as H-8′ and 7′-OCH_3_ to C-7′ that ascertained the position of the hydroxyl on C-2 and the methoxyl on C-7′. The remaining carbon (C-7), because of its proton-free environment, didn’t allow any HMBC correlations and was thus directly assigned based on the ^13^C chemical shift of C-7 (δ_C_ 153.5) which favors a hydroxyl substitution. The two monomers were connected through a C-8-C-3′ linkage as indicated by HMBC correlations from H-4′ to C-8 and nOe correlations between H-4′ and H-9 (Figure 3).

This was also supported by the downfield shift in the ^13^C-NMR signal of C-8 and C-3′ as compared to their respective monomeric units (113.0 to 121.0 ppm for nudol (**14**) and 113.3 to 120.6 ppm for lusianthridin (**21**)). The optical rotation of compound **4** was zero, and no Cotton effects was observed in the CD spectrum, inferring that **4** is a racemic mixture. On the basis of the above data, **4** was identified as 3,4,7′-trimethoxy-9′,10′-dihydro-[1,3′-biphenanthrene]-2,2,7,7′-tetraol and named lusidol A.

Compound **5** was obtained as a pale yellow amorphous powder. Its molecular formula was determined as C_31_H_26_O_7_ by the HRESIMS [M + H]^+^ peak at *m*/*z* 511.1740 (calcd. for C_31_H_27_O_7_ 511.1751) which indicated that it has the same molecular formula as compound **4**. The UV and IR spectra were identical to those of **4**. Comparison of the NMR spectral data of these two compounds revealed their structural similarities, suggesting that the structure of **5** comprises the same phenanthrene (nudol) and 9,10-dihydrophenanthrene (lusianthridin) moieties (Table 3). The only difference was observed in the linkage that connects the two units: in **6** the connection was via a C-1-C-3′ linkage as opposed to the C-8-C-3′ linkage in **4** (Figure 3). This was confirmed by the presence of ^1^H signal observed at δ_H_ 7.21 (d, *J* = 2.7 Hz, H-8) in **5**. The NOESY correlations from H-8 to H-9, H-10 to H-4′, as well as subsequent HMBC correlations from H-10 and H-4′ to C-1; H-8 to C-4b, C-6 and C-9 confirmed the position of this linkage. The optical rotation experiment result was zero, thus suggesting that **5** is a racemic compound. Therefore, compound **5** was identified as a 3,4,7′-trimethoxy-9′,10′-dihydro-[1,3′-biphenanthrene]-2,2′,5′,7-tetraol and named lusidol B.

**Figure 4:**
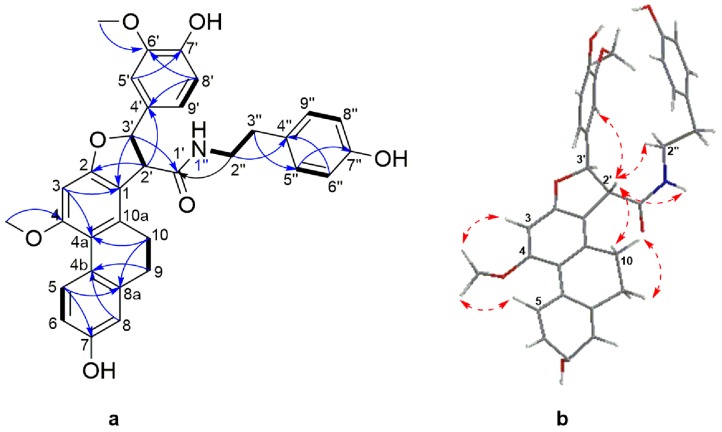
(**a**) Selected COSY (plain bonds) and HMBC (blue arrows) correlations of **6** (**b**) Energy minimized 3D structure and selected NOESY correlations (red dashed arrows) of **6**.

Compound **6** was obtained as a white amorphous powder. Its molecular formula was determined to be C_33_H_32_NO_7_ based on its HRESIMS [M + H]^+^ molecular ion peak at *m*/*z* 554.2169 (calcd for C_33_H_32_NO_7_ 554.2173). Its UV spectrum showed maximal absorption bands at 208, 281, 305 and 318 nm, indicative of a dihydrophenanthrene derivative. Its IR spectrum exhibited absorption bands at 3241 cm^−1^ (hydroxyl), 1704 and 1519 cm^−1^ (amide), 1199, 1118, 1015, 950, 814 and 773 cm^−1^ (aromatic rings). Analysis of the ^13^C-NMR and DEPT-135 spectra of **6** disclosed the presence of signals belonging to 24 aromatic carbons, comprising of eleven methine, seven quaternary, and six oxygenated tertiary carbons. The ^1^H-NMR spectrum (Table 3) showed resonances for three aromatic protons as an ABX system owing to a 9,10-dihydrophenanthrene moiety at δ_H_ 8.04 (1H, d, *J =* 9.4 Hz, H-5), 6.68 (1H, dd, *J* = 9.4, 2.5 Hz, H-6), 6.68 (1H, d, *J* = 2.5 Hz, H-8) and one aromatic proton singlet at δ_H_ 6.54 (1H, s, H-3). Two methylene proton signals at δ_H_ 2.45–2.55 (2H, m, H-9) and 2.55–2.60 (2H, m, H-10) and the corresponding carbon signals at δ_C_ 30.7 and 27.5, respectively, were characteristic of the methylene groups of a 9,10-dihydrophenanthrene skeleton. A 4′-hydroxy-3′-methoxyphenyl moiety comprising of three aromatic protons at δ_H_ 6.82 (1H, dd, *J* = 8.3, 1.5 Hz, H-5′), 6.82 (1H, d, *J* = 8.3 Hz, H-6′) and 6.99 (1H, d, *J* =1.5 Hz, H-9′) that formed the second ABX system. The presence of an A_2_B_2_ system characteristic of a symmetrical hydroxybenzyl ring was inferred by the resonance at δ_H_ 7.01 (2H, d, *J =* 8.4 Hz, H-5′′/9′′), 6.73 (2H, d, *J =* 8.4 Hz, H-6′′/8′′), as well as signals attributed to an ethylamide moiety at δ_H_ 3.46 (1H, td, *J* = 7.1, 5.5 Hz, H-2′′), 2.72 (1H, brt, *J* = 7.1 Hz, H-3′′), 7.28 (1H, t, *J* = 7.28, 1′-NH) and a amide carbonyl at δ_C_ 172.3, thus achieving a hydroxybenzylethylamide moiety. The connection between the three partial structures of 9,10-dihydrophenanthrene, 4′-hydroxy-3′-methoxyphenyl and hydroxybenzylethylamide moiety was achieved through a central furan ring as observed by the presence of two coupled methines at δ_H_ 4.11 (1H, d, *J* = 6.6 Hz, H-2′) and the second being oxygenated at δ_H_ 5.67 (1H, d, *J* = 6.6 Hz, H-3′). This was confirmed by extensive analysis of HMBC and NOESY spectra (Figure 4). The oxymethine proton at H-3′ (δ_H_ 5.67) showed HMBC correlations to C-2, C-4′, C-5′, C-9′ and the amide carbonyl at C-1′; H-2′ to C-1, C-2, C-1′, C-3′, and C-4′. Additional HMBC correlations were observed from the two aromatic protons at H-5′ to C-3′ and H-9′ to C-3′.

This was further supported by NOESY correlations between H-2′ to H-10, H-5′, H-9′ and 1′′-NH; H-3′ to H-5′, H-9′, and 1′′-NH. The assignments and locations of the two methoxyl groups at δ_H_ 3.88 (3H, s, 4-OCH_3_) and 3.82 (3H, s, 8′-OCH_3_) as well as the three hydroxyl groups on position C-7, C7′ and C-7′′ were confirmed by HMBC and NOESY analysis and supported by the characteristic chemical shift of the carbon bearing the groups. The hydroxyl bearing carbon position was settled in C-7 based on the HMBC correlation between H-5 and C-7 and by the characteristic chemical shift at δ_C_ 156.2. The positioning of the methoxyl on C-4 was affirmed from the NOESY cross peaks between H-3, H-5 and 4-OCH_3_.

The relative configuration of the methine protons H-2′ and H-3′ on the furan ring was defined as *trans*- based on NOESY correlations between H-2′ and H-5′′/9′′, suggesting these protons are on the same side of the furan ring. This was also supported by the coupling constant of 6.6 Hz, typical of *trans*-dihydrophenanthrenofuran derivatives [55,56], which allows the possibility of two enantiomeric stereoconfigurations, either (2′*R*, 3′*R*) or (2′*S*, 3′*S*). The absolute configurations of compound **6** could not be ascertained as its optical rotation was zero, suggesting a racemization of the *trans* form. The 3D structure of **6** (Figure 4) obtained from a minimized energy MM2 algorithm suggested that a *S*,*S*-*trans* configuration was more appropriate for this compound. Thus, the structure of **6** should be 7-hydroxy-2-(4-hydroxy-3-methoxyphenyl)-*N*-(4-hydroxyphenethyl)-10-methoxy-2,3,4,5-tetrahydrophenanthro[2,1-*b*]furan-3-carboxamide, and it was named moupilonin.

Compound **7** was obtained as a white amorphous powder. Its molecular formula was determined as C_26_H_26_O_7_ as deduced from its HRESIMS [M + H]^+^ ion peak at *m*/*z* 451.1753 (calcd. for C_26_H_27_O_7_ 451.1751), suggesting the presence of fourteen degrees of unsaturation. The UV spectrum showed absorption maxima at 210, 274 and 284 nm, suggesting that compound **7** has a dihydrophenanthrene skeleton. The IR absorption bands at ν_max_ at 3303, 1601, 1452 and 773 cm^−1^ were characteristic to a hydroxyl and aromatic moieties.

**Figure 5:**
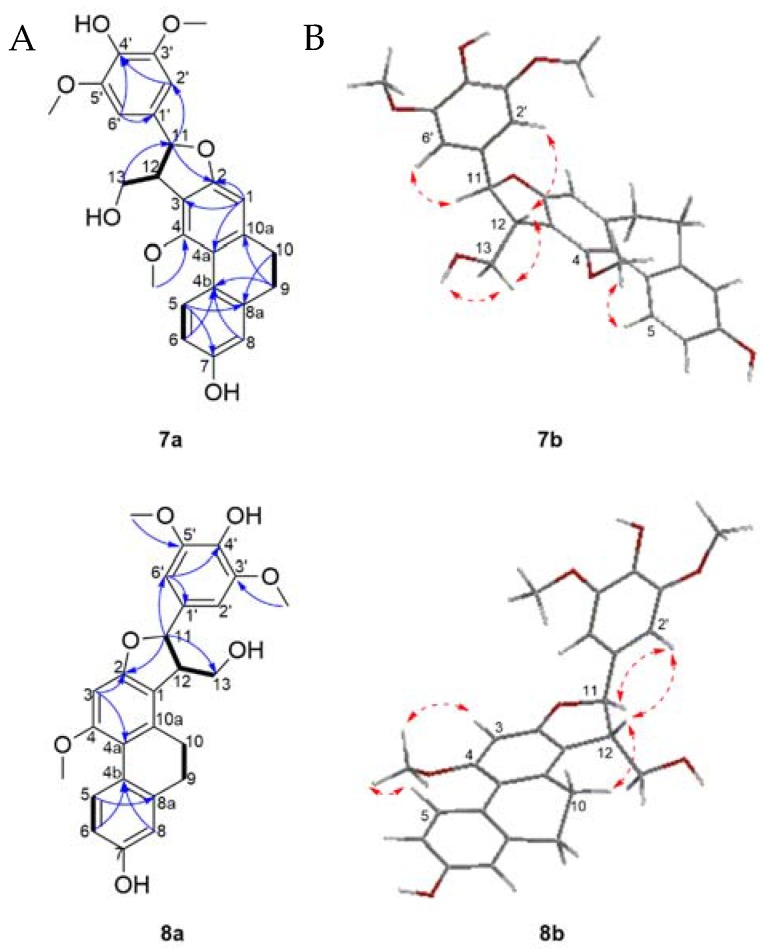
(**A**) Selected COSY (plain bonds) and HMBC (blue arrows) correlations of compounds **7**–**8**; (**B**) Energy minimized 3D structures and NOESY correlations (red dashed arrows) of compounds **7**–**8**.

The ^1^H-NMR spectrum (Table 4) showed resonances for four aromatic protons at δ_H_ 8.03 (1H, d, *J* = 9.2 Hz, H-5), 6.73 (1H, dd, *J* = 9.2, 2.8 Hz, H-6), 6.73 (1H, d, *J* = 2.8 Hz, H-8), and 6.56 (1H, s, H-1), as well as two methylenes at δ_H_ 2.69 (2H, m, H-9) and 2.67 (2H, m, H-10), which corresponds to resonances attributed to a 9,10-dihydrophenanthrene ring. A singlet aromatic proton integrating for two protons at δ_H_ 6.75 (2H, s, H-2′/H-6′) suggested the presence of a symmetrical 1′,3′,4′,5-tetrasubstituted aromatic ring. Three methoxy groups at δ_H_ 3.80 (6H, s, 3′/5′-OCH_3_), 3.60 (3H, s, 4-OCH_3_) and two hydroxyl groups at δ_H_ 8.29 (1H, s, 7-OH) and 7.24 (1H, s, 4′-OH), were also noticed. The linkage between the 9,10-dihydrophenanthrene unit and 1′,3′,4′,5-tetrasubstituted aromatic moiety of compound **7** was achieved through a furan ring as supported by signals assigned to an oxymethine proton at δ_H_ 5.66 (1H, d, *J* = 5.4 Hz, H-11), a methine at δ_H_ 3.73 (1H, m, H-12) and an oxygenated methylene at δ_H_ 3.83 (1H, m, H-13) and δ_H_ 4.08 (1H, ddd, *J* = 10.4, 4.7, 4.2 Hz, H-13) which coupled to an hydroxyl group at δ_H_ 4.17 (1H, dd, *J* = 6.1, 4.7 Hz, 13-OH). Analysis of the COSY spectrum (Figure 5) validated the OH-CH_2_(13)-CH(12)-CH(11) proton spin systems. Furthermore, HMBC correlations from H-11 to C-2, C-2′/6′; H-2′/6′ to C-11 as well as NOESY cross peaks between H-11 and H-2′/6′; H-2′/6′ and H-12 supported the structure as shown, connecting the phenyl to the furan ring at C-11.

The assignment of the substitution pattern was achieved based on HMBC correlations from 4-OCH_3_ to C4 and 3′/5′-OCH_3_ to C-3′/5′ suggested that the methoxy groups were attached to C-4 and C-3′/5′, respectively. NOESY correlations between 4-OCH_3_ and H-5, and between H-2′/H-6′ to 3′/5′-OCH_3_ also validated this assignment. The hydroxyl groups were assigned to positions C-7 and C-4′ based on HMBC long range correlations between 7-OH to C-7 (and additional correlation to C6 and C8), and 4′-OH to C-4′ (and additional correlations to C3′/5′). The relative stereoconfiguration of **7** was established on the basis of NOESY experiments, which displayed cross peaks between H-12 on the furan ring and H-2′/6′ indicated that both protons reside on the same side. In addition, the coupling constant of 5.4 Hz between H-11 and H-12 was in agreement with the reported literature values for the relative *trans*-configuration [57]. The absolute configurations of C-11 and C-12 were confirmed by CD determination: a negative Cotton effect at 284 nm suggested a 11*S*,12*R* form, which is consistent with previous reports [58,59]. The 3D structure was generated with an optimized energy minimization procedure using MM2, and suggested also a *trans* form as a 11*S*,12*R*. Therefore compound **7** was identified as (+)-(9*S*,10*R*)-9-(4-hydroxy-3,5-dimethoxyphenyl)-10-(hydroxymethyl)-11-methoxy-5,6,9,10-tetrahydrophenanthro[2,3-*b*]furan-3-ol and named cyrtonesin A .

Compound **8** was obtained as a white amorphous powder and its molecular formula was established as C_26_H_26_O_7_ by the HREIMS peak at *m*/*z* 451.1748 [M + H]^+^ (calcd. for C_26_H_27_O_7_ 451.1751). The mass, UV, IR and NMR spectra (Table 4) indicated the striking similarity between compounds **7** and **8**. The difference between the two compounds is the positioning of the furan ring on the dihydrophenanthrene chore. In compound **7**, the furan ring was positioned as -C2-C3-C12-C-11-O-, while in compound **8** it is positioned as a -C-1-C2-O-C-11-C-12-. This was corroborated by the presence of a singlet proton resonance at δ_H_ 6.56 (1H, s, H-3) as well as HMBC correlations (Figure 5a) from H-3 to C-2 and C-4a as well as NOESY correlations (Figure 5b) between 4-OCH_3_, H-3 and H-5 also validated the proton in position C-**3**.

The methylene at position C-13 also had NOESY correlation with the proton H-10 on the dihydrophenanthrene subunit. The relative configuration between H-11 and H-12 was assumed to be *trans* because of the NOESY correlation between H-12 to H-2′/H-6′ as well as the coupling constant of 3.3 Hz supporting a *trans*-configuration as previously reported in dihydrophenanthrenofuran derivatives [54]. The CD spectrum of **8** showed a negative Cotton effect at 273 nm, allowing the assignment of a 11*S*, 12*R*. Based on the above evidence, the structure of compound **8** was determined to be (+)-(2*S*,3*R*)-2-(4-hydroxy-3,5-dimethoxyphenyl)-3-(hydroxymethyl)-10-methoxy-2,3,4,5-tetra-hydrophenanthro[2,1-*b*]furan-7-ol. Compound **8** was named cyrtonesin B.

## 3. Experimental Section

### 3.1. General Procedures

Optical rotations were measured with a P-2000 polarimeter (Jasco, Lisses, France) and circular dichroïsm spectra were recorded on a Jasco J-510 spectropolarimeter apparatus (Jasco). UV spectra were recorded on a UV-2401 PC spectrometer (Shimadzu, Kyoto, Japan). IR spectra were obtained on a 380 FT-IR spectrophotometer (Thermo Electron Corporation, Saint-Herblain, France).The 1D and 2D NMR spectra were recorded on a Bruker 500 MHz Avance III spectrometer (Bruker BioSpin, Rheinstetten, Germany) equipped with a DCH ^13^C/^1^H Cryoprobe (Bruker Biospin, Fallanden, Switzerland). Acetone-*d_6_* and methanol-*d_4_* (Euriso-Top, Saint-Aubin, France) were used as deuterated solvents and their protonated residual signals were used as internal standard at 2.05 ppm and 3.31 ppm respectively, relative to TMS. The HRESIMS analysis were performed on a HPLC-DAD/UV-MS Agilent 1200 series coupled to a 6520 Q-ToF mass spectrometer (Agilent Technologies, Santa Clara, CA, USA). The acquisition of mass spectra was conducted in ESI positive ion mode. Column chromatography and vacuum liquid chromatography were carried out using 40–60 µm silica gel (Sigma Aldrich, St-Louis, MO, USA). The obtained fractions were monitored by TLC and the spots were visualized under UV light (254 nm) and by 2% sulfuric vanillin reagent. Sephadex LH-20 (Sigma Aldrich, St-Louis, MO, USA), was used for gel chromatography. RP-HPLC experiments were conducted on a Gilson LC system (Gilson Inc., Limburg an der Lahn, Germany) equipped with a semi preparative Kinetex Axia C-18 column (100 mm × 21.2 mm i.d, 5 µm; Phenomenex, Torrance, CA, USA). Experiments were conducted at a wavelength of 280 nm. Preparative TLC was performed on a glass supported silica gel 60F254 (0.25 mm thickness; Merck, Darmstadt, Germany). Analytical grade solvents of HPLC quality were purchased from Sigma Aldrich.

### 3.2. Plant Material

Fifteen fresh specimens of *C. paniculatum* (Ruiz & Pav.) Garay were purchased from the orchid farm Orquidea del Valle, Ginebra, Colombia in October 2013 and imported to France, according to the Convention of Natural Trades in Endangered Species (CITES). The voucher specimens (No 58054 and 58056) were deposited at the Herbarium of CUVC, Universidad del Valle, Cali, Colombia.

### 3.3. Extraction and Isolation

The pseudobulbs (7.5 kg) were cut into small pieces, ground and soaked in demineralized water for 30 min to get rid of the mucilage content. The remaining part was then macerated with ethanol and the filtrates were evaporated under reduced pressure to obtain a crude ethanolic extract (60 g). The ethanolic extract was suspended in water and successively partitioned with cyclohexane, CH_2_Cl_2_ and *n*-BuOH to yield after solvent removal a cyclohexane extract (0.60 g), a CH_2_Cl_2_ extract (1.67 g) and an *n*-BuOH extract (7.88 g). The CH_2_Cl_2_ extract was subjected to silica column chromatography (cyclohexane/EtOAc; 100:0 to 0:100, EtOAc/CH_3_OH 100:0 to 0:100) to afford 24 fractions (A–X). Fraction D (24.4 mg) was subjected to preparative thin layer chromatography (PTLC; CHCl_3_/CH_3_OH 94:6) to afford compound **9** (6.3 mg). Fraction F (51.6 mg) was fractionated on Sephadex LH-20 (CH_3_OH) to obtain sub-fractions F1-F8. Sub-fractions F4 (7.4 mg) and F5 (25.7 mg) were further purified using PTLC (CHCl_3_/CH_3_OH 94:6) to afford **10** (4 mg), **11** (5 mg), **12** (5 mg) and **13** (0.9 mg). Fraction G (165.4 mg) was purified using Sephadex LH-20 (CH_3_OH) to obtain eight sub-fractions (G1–G8). Sub-fraction G8 was obtained as a pure compound **14** (2.8 mg). Fraction H (71.6 mg) was also subjected to Sephadex LH-20 (CH_3_OH) to obtain four sub-fractions (H1–H4). H1 (12.2 mg) led to the isolation of **15** (1.4 mg) and **16** (1.3 mg) on PTLC eluting with CHCl_3_/CH_3_OH (94:6). Sub-fraction H2 (4.7 mg) led to the isolation of **17** (1 mg) and **18** (0.9 mg) using PTLC (CHCl_3_/CH_3_OH). Fraction I (87 mg) was purified on Sephadex LH-20 (CH_3_OH) to obtain seven sub-fractions (I1-I7). Sub-fraction I1 (25.6 mg) led to the isolation of **19** (13.1 mg), while sub-fraction I2 (13.3 mg) afforded compounds **20** (5.3 mg) and **21** (4.1 mg) using PTLC eluting with CHCl_3_/CH_3_OH (94:6). Sub-fraction I7 (4.2 mg) was purified on a PTLC (CHCl_3_/CH_3_OH; 94:6) to obtain compound **1** (1.3 mg). Fraction J (32.2 mg) was fractioned on Sephadex LH-20 (CH_3_OH) and PTLC (CHCl_3_/CH_3_OH; 94:6) to obtain **22** (0.9 mg) and **23** (1 mg). Fraction K (29.5 mg) was purified on Sephadex LH-20 (CH_3_OH) affording 7 sub-fractions (K1–K7). Sub-fraction K6 (4.7 mg) was subjected to a PTLC eluting with CHCl_3_/CH_3_OH (94:6) affording compounds **3** (0.6 mg), **24** (2 mg) and **25** (0.6 mg). Fraction L (26.6 mg) was also subjected to Sephadex LH-20 (CH_3_OH) to yield 8 sub-fractions (L1–L8). Sub-fraction L5 (2.2 mg) was purified using PTLC with CHCl_3_/CH_3_OH (94:6) as the eluting solvent to yield **26** (0.6 mg). Fraction M (90.0 mg) was subjected to Sephadex LH-20 and eluted with CH_3_OH to afford 10 sub-fractions (M1–M10). Compounds **27** (2.4 mg) and **28** (6.7 mg) were obtained from sub-fraction M2 (19.9 mg) by PTLC using CHCl_3_/CH_3_OH as the mobile phase. Sub- fraction M7 (13.2) was further purified by PTLC using CHCl_3_/CH_3_OH (94:6) to obtain **29** (2.1 g) and **30** (2.6 mg). Sub-fraction M8 (2.9 mg) was passage over PTLC that was eluted with CHCl_3_/CH_3_OH (94:6) to yield compound **31** (1.1 mg). Sub-fraction M9 (11.6 mg) was purified by PTLC with CHCl_3_/CH_3_OH (94:6) to yield compounds **4** (0.7 mg), **5** (0.6 mg) and **32** (0.9 mg). Fraction O (71.7 mg) was subjected to Sephadex LH-20 eluting with CH_3_OH, to obtain 5 sub-fractions (O1–O5). Sub-fraction O3 (9.8 mg) was purified by PTLC with CHCl_3_/CH_3_OH (94:6) to obtain compound **2** (2.8 mg). Sub-fraction O5 (2.4 mg) was purified by PTLC with CHCl_3_/CH_3_OH (94:6) to give compound **33** (0.7 mg). Fraction P (63 mg) was fractionated on Sephadex LH-20 (CH_3_OH) to obtain six sub-fractions (P1–P6). Sub-fraction P4 (3.7 mg) was further subjected to PTLC eluting with CHCl_3_/CH_3_OH (94:6) to obtain compounds **6** (0.6 mg), **7** (0.9 mg) and **8** (0.6 mg). Fraction Q (19.5 mg) was purified on Sephadex LH-20 (CH_3_OH) affording six sub-fractions. Compounds **34** (3 mg) and **35** (0.8 mg) were obtained from sub-fraction Q4 (7 mg) through a PTLC with CHCl_3_/CH_3_OH (94:6) as the eluting solvent.

Fresh leaves were lyophilized and grinded to afford 70 g of dried powder and were successively extracted with cyclohexane, CH_2_Cl_2_ and CH_3_OH (1 g/15 mL × 3) to afford a cyclohexane extract (6.99 g), a CH_2_Cl_2_ extract (1.23 g) and a CH_3_OH extract (4.98 g). The CH_2_Cl_2_ extract was subjected to vacuum liquid chromatography (VLC) using cyclohexane, EtOAc and CH_3_OH to afford six main fractions (A–F). Fraction E (98 mg) was subjected to semi-preparative HPLC (40%–45% B in 5 min, 45% isocratic mode for 30 min, B = CH_3_OH/0.05% formic acid, A = H_2_O/0.05% formic acid) to afford compounds **13** (3.5 mg), **18** (0.7 mg), **19** (1 mg), and **21** (1.7 mg). Fractions F (158.4 mg) was also subjected to semi- preparative RP-HPLC (35% B for 35 min) to afford compound **36** (9.6 mg).

### 3.4. Compound Characterization

*Cyrtopodin* (**1**). Brown amorphous powder (1.3 mg); UV (CH_3_OH) λ_max_ (log ε): 216 (3.96), 261 (4.38), 281 (4.02), 312 (3.48), 344 (2.52); IR (FT-IR) ν_max_: 3370, 2935, 2831, 1616, 1573, 1465, 1268, 1118, 1061, 1021, 953, 860, 770 cm^−1^; ^1^H-NMR and ^13^C-NMR see Table 1; HRESIMS: *m*/*z* 331.1188 [M + H]^+^ (Calcd. for C_18_H_19_O_6_, 331.1176).

*(+)-9S-Hydroxyerianthridin* (**2**). White amorphous powder (2.8 mg); ${\left[\alpha \right]}_{D}^{25}$ + 6.4 (c 0.09, CH_3_OH); CD (CH_3_OH): nm (Δε): 236.2 (−20.69), 280.9 (+5.39); (UV (CH_3_OH) λ_max_ (log ε): 220 (4.01), 262 (3.91), 282 (4.00), 348 (2.28), 364 (2.26); IR (FT-IR) ν_max_: 3227, 2936, 2834, 1611, 1584, 1446, 1412, 1348, 1213, 1112, 1066, 999, 946, 870, 828 cm^−1^; ^1^H-NMR and ^13^C-NMR see Table 1; HRESIMS: *m*/*z* 311.0884 [M + Na]^+^; (Calcd. for C_16_H_16_O_5_Na, 311.8900).

*Gastrodiconfusarin* (**3**). Yellow amorphous powder (0.6 mg); UV (CH_3_OH) λ_max_ (log ε): 212 (4.11), 268 (4.09), 289 (3.63), 297 (4.00), 301 (3.59), 314 (3.45); IR (FT-IR) ν_max_: 3336, 2931, 2936, 1606, 1512, 1454, 1352, 1221, 1106, 1017, 822, 773 cm^−1^; ^1^H-NMR and ^13^C-NMR see Table 1; HRESIMS *m*/*z* 407.1508 [M + H]^+^ (Calcd. for C_24_H_23_O_6_, 407.189).

*Lusidol A* (**4**). Pale yellow amorphous powder (0.7 mg); ${\left[\alpha \right]}_{D}^{25}$ 0 (c 0.04, CH_3_OH); UV (CH_3_OH) λ_max_ (log ε): 213 (4.80), 263 (4.82), 296 (4.47), 310 (4.40), 349 (3.58), 367 (3.55); IR (FT-IR) ν_max_: 3245, 2931, 2835, 1608, 1585, 1451, 1393, 1351, 1210, 1162, 1118, 1076, 999, 948, 865, 830 cm^−1^; ^1^H-NMR and ^13^C-NMR see Table 2; HRESIMS: *m*/*z* 511.1742 [M + H]^+^ (Calcd. for C_31_H_27_O_7_, 511.1751).

*Lusidol B* (**5**). Pale yellow amorphous powder (0.6 mg); ${\left[\alpha \right]}_{D}^{25}$ 0 (c 0.04, CH_3_OH); UV (CH_3_OH) λ_max_ (log ε): 212 (4.72), 264 (4.66), 297 (4.37), 309 (4.29), 350 (3.28), 367 (3.27). IR (FT-IR) ν_max_: 3271, 2931, 2835, 1608, 1585, 1451, 1393, 1351, 1210, 1162, 1118, 1076, 999, 948, 865, 830; 527 cm^−1^; ^1^H-NMR and ^13^C-NMR see Table 2; HRESIMS: *m*/*z* 511.1740 [M + H]^+^ (Calcd. for C_31_H_27_O_7_, 511.1751).

*(±)-Moupilonin* (**6**). White amorphous powder (0.9 mg); ${\left[\alpha \right]}_{D}^{25}$ 0 (c 0.07, CH_3_OH); UV (CH_3_OH) λ_max_ (log ε): 208 (4.37), 281 (3.97), 305 (3.73), 318 (3.51); IR (FT-IR) ν_max_ : 3241, 2935, 2829, 1704, 1519, 1447, 1349, 1237, 1199, 1118, 1015, 950, 814, 773 cm^−1^; ^1^H-NMR and ^13^C-NMR see Table 3; HRESIMS: *m*/*z* 554.2169 [M + H]^+^ (Calcd. for C_33_H_32_NO_7_, 554.2173).

*(+)-Cyrtonesin A* (**7**). White amorphous powder (0.6 mg); ${\left[\alpha \right]}_{D}^{25}$ 27 (c 0.04, CH_3_OH); CD (CH_3_OH): nm (Δε): 233.1 (+0.84), 284.2 (−0.95); UV (CH_3_OH) λmax (log ε): 210 (4.30), 274 (3.73), 284 (3.76); IR (FT-IR) ν_max_: 3303, 2935, 2831, 1601, 1452, 1359, 1239, 1217, 1114, 1068, 1033, 1008, 977, 951, 824, 773 cm^−1^; ^1^H-NMR and ^13^C-NMR see Table 4; HRESIMS: *m*/*z* 451.1753 [M + H]^+^ (Calcd. for C_26_H_27_O_7_ 451.1751).

*(+)-Cyrtonesin B* (**8**). White amorphous powder (0.6 mg); ${\left[\alpha \right]}_{D}^{25}$ 26 (c 0.02, CH_3_OH); CD (CH_3_OH): nm (Δε): 235.0 (+1.11), 272.8 (−7.26); UV (CH_3_OH) λ_max_ (log ε): 207 (4.29), 292 (3.73), 301 (3.77); IR (FT-IR) ν_max_: 3320, 2936, 2825, 1599, 1460, 1354, 1239, 1221, 1060, 1035, 1008, 977, 955, 825, 773 cm^−1^; ^1^H-NMR and ^13^C-NMR see Table 4; HRESIMS: *m*/*z* 451.1748 [M + H]^+^ (Calcd. for C_26_H_27_O_7_ 451.1751).

## 4. Conclusions

Due to extreme environmental and territorial conditions as well as predation, plant species have evolved to produce complex secondary metabolites as a means of survival. In orchids, stilbenoids and phenanthrene derivatives are produced as major phytoalexins produced in response to various biotic and abiotic stressors, mostly fungal, bacterial and worm attacks [60,61,62,63,64,65,66]. The phytochemical study of CH_2_Cl_2_ extract from the aerial parts of *C. paniculatum* led us to the isolation of an long list of polyphenolic derivatives, mainly stilbenoids. Monomeric stilbenoid derivatives, comprising bibenzyls, 9,10-dihydrophenanthrenes, phenanthrenes and phenanthrenequinones, are well represented in this orchid. The second set of isolated compounds consisted of phenolic adducts (4-hydroxybenzyl) coupled to a 9,10-dihydrophenanthrene or phenanthrene derivative. This type of 4-hydroxybenzyl adduct (gastrodigenin) is very common in orchids and mostly found in the genus *Arundina* [67,68], *Bletilla* [69,70,71,72] and *Pleione* [40,73,74]. The third set of isolated compounds was a combination of a 9,10-dihydrophenanthrene and a phenylpropane derivative (*trans*-feruloyltyramine for **6**, synapyl alcohol for **7** and **8**). The connection between the two units allows a new cyclization leading to a furan ring. It is noteworthy that this kind of dihydrophenanthrene derivative is considered as a chemotaxonomic marker for the species in the genus *Pleione* [59,73,75,76,77], but has been also found in the genus *Bulbophyllum* [55] and *Cremastra* [54]. The last set of compounds were the dimers, occurring as either homo- (two units of 9,10-dihydrophenanthrene or phenanthrene) or as heterodimers (combinations of a 9,10-dihydrophenanthrene and a phenanthrene unit) having a C-C bond linkage. These biphenanthrene derivatives occur mostly in species of the genus *Bletilla* [33,41,71,78,79,80], *Bulbophyllum* [55,81,82,83] and *Cremastra* [54,84,85,86,87,88]. The existence of dimers together their respective monomers in the same species provides support for a proposed biogenetic pathway for biaryl-derived compounds occurring from their corresponding monomers as a result of free radical [56] or an enzymatic oxidative phenolic coupling reaction [82,89,90,91]. This study reveals for the first time the chemical diversity of phenolic constituents produced by an endemic South-American orchid, *Cyrtopodium paniculatum*.

## Figures and Tables

**Table 1:** ^1^H (500 MHz) and ^13^C (125 MHz), HMBC and NOESY spectroscopic data of compounds **1**–**3** (δ in ppm, *J* in Hz) in acetone-*d*_6_.

No.	1				2				3			
	δ_H_	δ_C_	HMBC	NOESY	δ_H_	δ_C_	HMBC	NOESY	δ_H_	δ_C_	HMBC	NOESY
1		142.5			6.59 (s)	112.7	2, 3, 4, 4a, 10	10, 10′		119.7		
2		138.5				149.9				147.9		
3		149.5				141.1				142.3		
4	8.90 (s)	103.9	2, 3, 4b, 10a	3/5-OCH_3_		152.1				150.6		
4a		124.2				120.2				127.8		
4b		118.8				124.3				125.5		
5		152.3			8.09 (d, 8.6)	129.2	4a, 7, 8, 8a, 9	4-OCH_3_, 6	9.21 (d, 9.4)	124.4	4a, 4b, 7	4-OCH_3_, 6
6		142.7			6.76 (dd, 8.6, 2.7)	114.6	4b, 7, 8	5	7.24 (d, 9.4)	118.2	4b, 7, 8	5
7		150.2				157.1				147.2		
8	7.17 (s)	109.7	4b, 5, 6, 7, 9	7-OH, 9	7.11 (d, 2.7)	112.8	4b, 6, 7, 9	9		142.1		
8a		131.0				143.3				128.8		
9	7.50 (d, 8.9)	125.3	4b, 8, 10a	8, 10	4.63 (dd, 10.6, 4.6)	68.9	4b, 8, 8a, 10a, 10	8, 10, 10′	7.87 (d, 9.5)	120.3	8, 4b, 10a	8-OCH_3_
10	7.88 (d, 8.9)	120.1		9, 1-OCH_3_	2.70 (dd, 14.3, 10.6)	40.1	1, 4a, 8a, 9, 10a	1, 9, 10	7.90 (d, 9.5)	124.3	1, 4a, 8a	2′/6′, 7′
10					2.83 (dd, 14.3, 4.6)	40.1	1, 4a, 8a, 9, 10a	1, 9, 10′				
10a		122.6	1, 4a, 8a			132.1				129.2		
1-OCH_3_	3.99 (s)	61.1	1	2-OH, 10								
3-OCH_3_	4.06 (s)	56.3	3	2-OH, 4	3.85 (s)	61.1	3		4.06 (s)	61.6	3	
4-OCH_3_					3.72 (s)	60.3	4		3.95 (s)	60.1	4	
5-OCH_3_	4.03 (s)	60.5	5	4								
6-OCH_3_	4.02 (s)	61.4	6	7-OH								
8-OCH_3_									3.90 (s)	61.5	8	
2-OH	7.91 (s)		1, 2, 3	1/3-OCH_3_								
7-OH	8.39 (s)		6, 7, 8	6-OCH_3_, 8								
9-OH					4.32 (brs)							
1′										133.0		
2′/6′									7.08 (d, 8.6)	130.1	3′/5′, 4′	3′/5′, 7′, 10
3′/5′									6.68 (d, 8.6)	115.9	2′/6′, 1′	2′/6′
4′/										156.3		
7′									4.41 (s)	30.9	1, 10a, 1′, 2′/6′	10

**Table 2:** ^1^H (500MHz) and ^13^C (125 MHz), HMBC and NOESY spectroscopic data of compounds **4**–**5** (δ in ppm, *J* in Hz) in acetone-*d*_6_.

No.	4				5			
	δ_H_	δ_C_	HMBC	NOESY	δ_H_	δ_C_	HMBC	NOESY
1	7.11 (s)	109.5	2, 3, 4a, 10	10		118.4		
2		150.0				148.1		
3		142.9				142.6		
4		152.4				151.4		
4a		119.4				119.4		
4b		124.8				124.5		
5	9.42 (d, 9.2)	128.1	4a, 7, 8a	4-OCH_3_, 6	9.42 (d, 9.2)	129.3	4a, 7, 8b	4-OCH_3_, 6
6	7.31 (d, 9.2)	117.7	4b, 8	5	7.20 (dd, 9.2, 2.7)	117.6	4b, 7, 8	5
7		153.5				156.1		
8		121.0			7.21 (d, 2.7)	112.2	4b, 6, 9	9
8a		133.5				134.5		
9	7.43 (s)	125.6	4b, 8, 10a	10, 4′	7.41 (d, 9.0)	126.6	4b, 8, 10a	8, 10
10	7.43 (s)	127.2	1, 4a, 8a	1, 9	7.44 (d, 9.0)	126.0	1, 4a, 8a	9, 4′
10a		130.1				129.8		
3-OCH_3_	4.01 (s)	61.4	3		4.06 (s)	61.3	3	
4-OCH_3_	4.00 (s)	60.2	4	5	4.00 (s)	60.1	4	5
1′	6.92 (s)	116.1	2′, 3′, 4a′, 10′	10′	6.89 (s)	115.7	2′, 3′, 4a′, 10′	10′
2′		154.6				154.3		
3′		120.6				121.1		
4′	8.28 (s)	133.2	8, 2′, 4b′, 10a′	9	8.27 (s)	133.0	1, 2′, 4b′, 10a′	10
4a′		126.2				126.1		
4b′		115.8				115.8		
5′		156.0				155.9		
6′	6.39 (d, 2.6)	101.6	4b′, 7′, 8′	7′-OCH_3_	6.38 (d, 2.6)	101.6	4b′, 7′, 8′	7′-OCH_3_
7′		159.5				159.4		
8′	6.42 (d, 2.6)	106.1	4b′, 6′, 7′, 9′	7′-OCH_3_, 9′	6.42 (d, 2.6)	106.1	4b′, 6′, 7′, 9′	7′-OCH_3_, 9′
8a′		141.5				141.5		
9′	2.80 (brs)	30.7	4b′, 8′, 10a′	8′	2.80 (brs)	30.7	4b′, 8′, 10a′	8′
10′	2.80 (brs)	31.6	1′, 4a′, 8a′′	1′	2.80 (brs)	31.7	1′, 4a′, 8a′′	1′
10a′		139.6				139.5		
7′-OCH_3_	3.73 (s)	55.4	7′	6′, 8′	3.74 (s)	55.4	7′	6′, 8′

**Table 3:** ^1^H (500 MHz), ^13^C (125 MHz), HMBC and NOESY spectroscopic data of compound 6 (δ in ppm, *J* in Hz) in acetone-*d*_6_.

No.	6			
	δ_H_ (*J*, Hz)	δ_C_	HMBC	NOESY
1		116.7		
2		160.4		
3	6.54 (s)	93.6	1, 2, 4, 4a	4-OCH_3_
4		159.2		
4a		117.6		
4b		125.7		
5	8.04 (d, 9.4)	130.0	4a, 8a, 7	4-OCH_3_, 6
6	6.68 (dd, 9.4, 2.5)	113.6	4b, 8	5
7		156.2		
8	6.68 (d, 2.5)	115.0	4b, 6, 9	9
8a		139.8		
9	2.55, 2.60 (m)	30.7	8	8, 10
10	2.44, 2.55 (m)	27.5	8a	9, 2′
10a		137.2		
4-OCH_3_	3.88 (s)	56.1	4	3, 5
1′		172.3		
2′	4.11 (d, 6.6)	58.6	1, 2, 1′, 3′, 4′	10, 3′, 5′, 9′, 1′′-NH
3′	5.67 (d, 6.6)	89.8	2, 1′, 2′, 4′, 5′, 9′	2′, 5′, 9′, 1′′-NH
4′		133.8		
5′	6.82 (dd, 8.3, 1.5)	119.5	3′, 7′, 9′	2′, 3′
6′	6.82 (d, 8.3)	115.9	4′, 8′	
7′		147.6		
8′		148.5		
9′	6.99 (d, 1.5)	110.3	3′, 5′, 7′	2′, 3′, 8′-OCH_3_
8′-OCH_3_	3.82 (s)	56.4	8′	9′
2′′	3.46 (td, 7.1, 5.5)	41.9	1′, 3′′, 4′′	1′′-NH, 3′′
3′′	2.72 (brt, 7.1)	35.5	2′′, 5′′/9′′	2′′
4′′		130.9		
5′′/9′′	7.01 (d, 8.4)	130.6	3′′, 9′′/5′′, 6′′	6′′/8′′
6′′/8′′	6.73 (d, 8.4)	116.1	4′′, 8′′/6′′	5′′/9′′
7′′		156.8		
1′′-NH	7.28 (t, 5.5)			2′, 3′, 2′′

**Table 4:** ^1^H (500MHz), ^13^C (125 MHz), HMBC and NOESY spectroscopic data of compounds **7**–**8** (δ in ppm, *J* in Hz) in acetone-*d*_6_.

No.	7				8			
	δ_H_ (*J*, Hz)	δ_C_	HMBC	NOESY	δ_H_ (*J*, Hz)	δ_C_	HMBC	NOESY
1	6.56 (s)	105.9	2, 3, 4a, 10			117.4		
2		160.5				160.3		
3		118.7			6.56 (s)	93.5	2, 4a	4-OCH_3_
4		156.0				158.9		
4a		120.9				116.8		
4b		125.4				125.8		
5	8.03 (d, 9.2)	128.9	7, 8a	4-OCH_3_, 6	8.04 (d, 9.3)	130.0	7, 8a	4-OCH_3_, 6
6	6.73 (dd, 9.2, 2.8)	114.2		5, 7-OH	6.68 (dd, 9.3, 2.7)	113.6	4b, 8	5
7		156.7				156.1		
8	6.73 (d, 2.8)	115.2		9, 7-OH	6.69 overlapped	115.0	4b, 6, 9	9
8a		140.2				139.8		
9	2.69 (m)	30.8	4b, 10, 10a	8	2.59-2.64 (m)	30,4		8
10	2.67 (m)	31.5	8a, 9, 1	1	2.59-2.71 (m)	27.5		12
10a		142.1				137.1		
4-OCH_3_	3.60	60.0	4	5, 12	3.87 (s)	56.0	4	3, 5
7-OH	8.29 (brs)		6, 7, 8	6, 8	8.22 (brs)			11, 12
11	5.66 (d, 5.4)	88.4	2, 13, 2′/ 6′	12, 13, 2′/6′	5.71 (d, 3.4)	88.2	2, 13, 2′	10, 11, 13, 2′/6′
12	3.73 (m)	54.3		11, 13, 2′/6′	3.54 (dt, 8.7, 3,4)	54.5		12
13	4.08 (ddd, 10.4, 4.7, 4.2)	63.5		12	3.63 (m)	64.6		12
	3.83 (m)			11, 12	3.90 (m)			-
13-OH	4,17 (dd, 6.1, 4.7)			13	4.22 (brt 5.3)			-
1′		133.9				134.4		
2′/6′	6.75 (s)	104.3	11, 1′, 4′, 6′/2′, 3′/5′	11, 12, 3′/5′-OCH_3_	6.69 (s)	103.9	11, 1′, 4′, 6′/2′, 3′/5′	11, 12, 3′/5′-OCH_3_
3′/5′		148.8				148.8		
4′		136.6				136.4		
3′/5′-OCH_3_	3.80 (s)	56.7	3′/5′	2′/6′	3.78 (s)	56.7	3′/5′	2′/6′
4′-OH	7.24 (brs)		3′/5′, 4′		7.23 (brs)		3′/5′	

## Supplementary Materials

Supplementary data associated with this article can be found, in the online version, at www.mdpi.com/1420-3049/21/10/1418/s1.
