# Supplementary material for: Chemical Constituents from the Aerial Parts of Cyrtopodium paniculatum
Source: Molecules. 2016 Oct 24;21(10):1418. doi: 10.3390/molecules21101418 (PMC6274024; doi:10.3390/molecules21101418)
Supplement: Supplementary file 1 [file molecules-21-01418-s001.pdf]

Supplementary Materials: Chemical Monstituents from the Aerial Parts of *Cyrtopodium paniculatum*

Florence Auberon, Opeyemi Joshua Olatunji, Gaëtan Herbette, Diamondra Raminoson, Cyril Antheaume, Beatriz Soengas, Frédéric Bonté and Annelise Lobstein

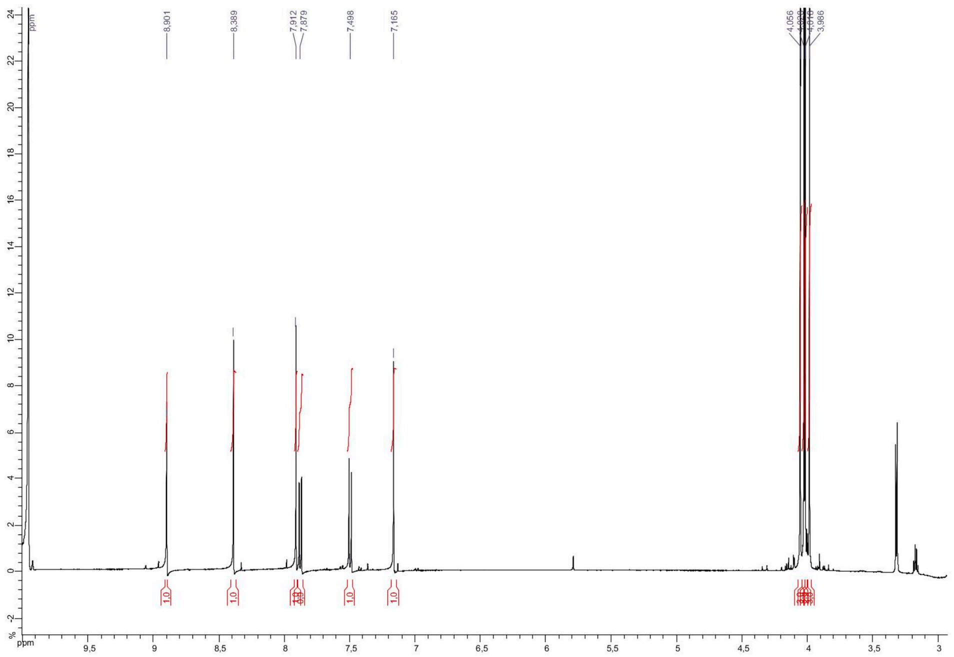

Figure S1. ^{1}H-NMR (500 MHz, acetone-d_6) spectrum of cyrtopodin (**1**).

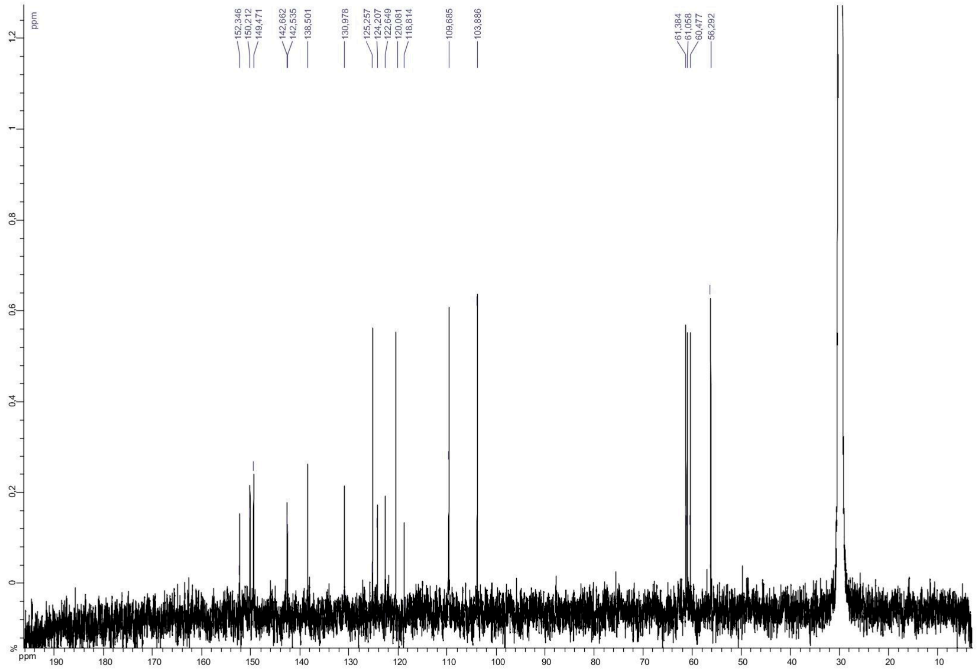

Figure S2. ^{13}C-NMR (125 MHz, acetone-d_6) spectrum of cyrtopodin (**1**).

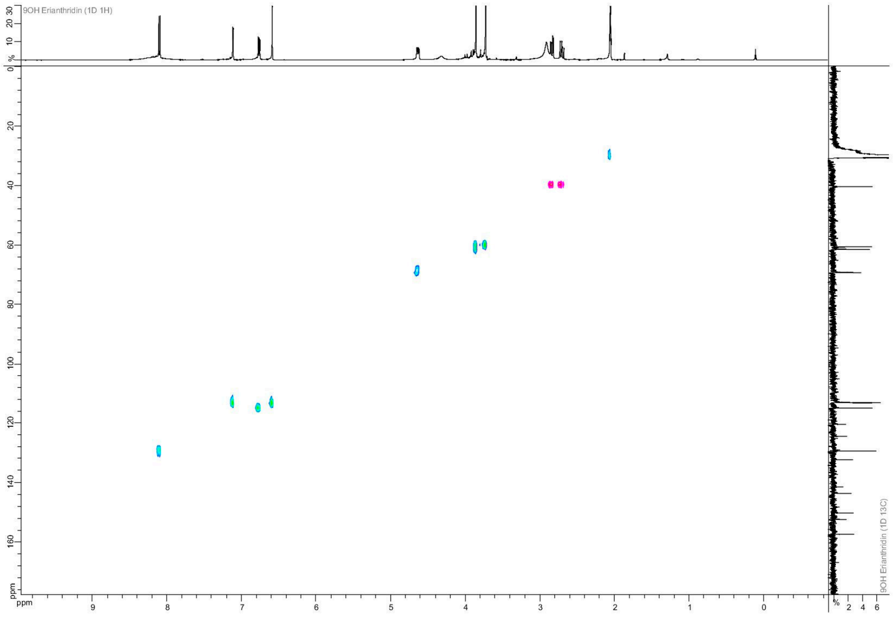

Figure S3. HSQC spectrum of cyrtopodin (**1**).

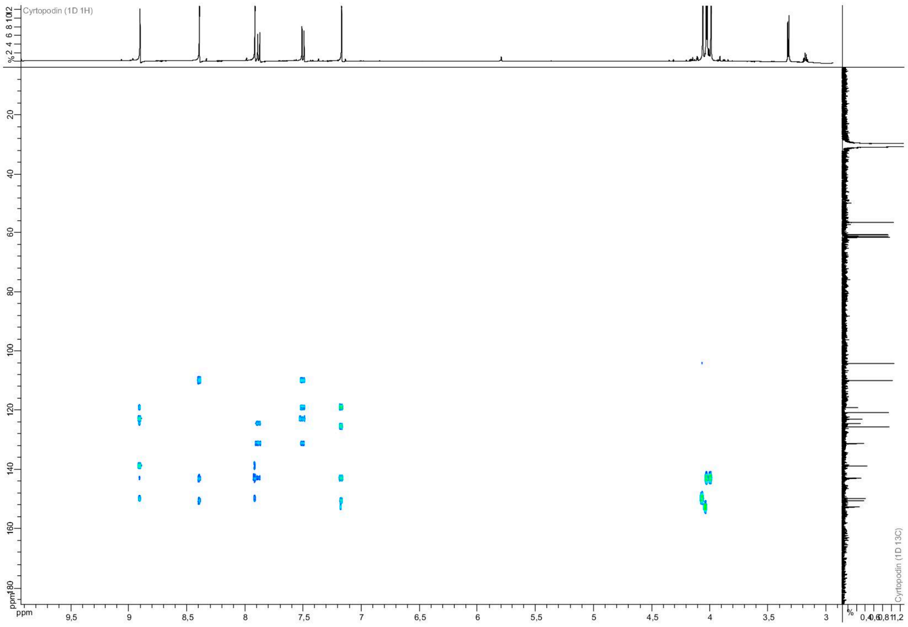

Figure S4. HMBC spectrum of cyrtopodin (**1**).

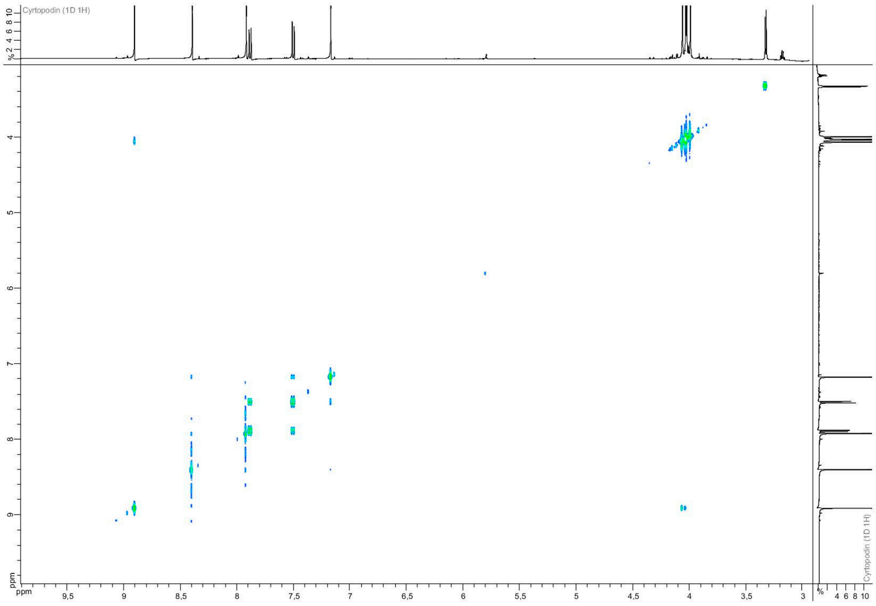

Figure S5. NOESY spectrum of cyrtopodin (**1**).

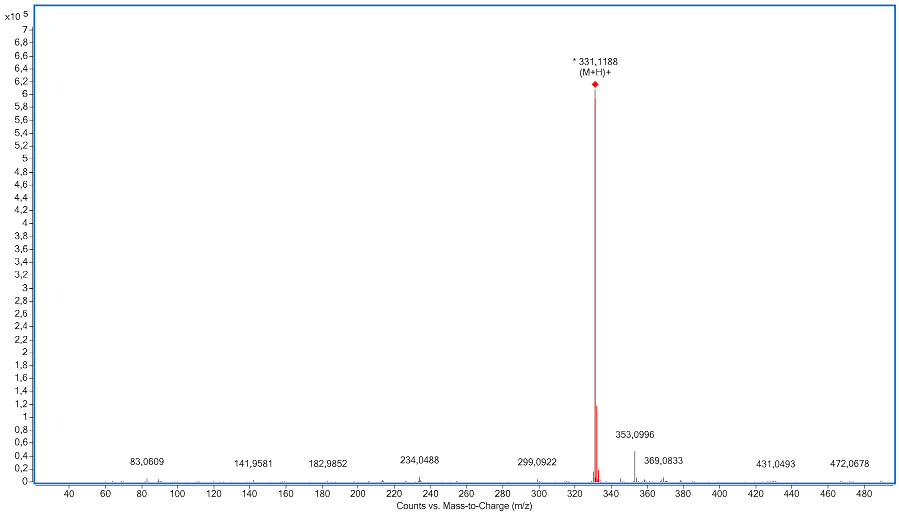

Figure S6. HRESIMS spectrum of cyrtopodin (**1**).

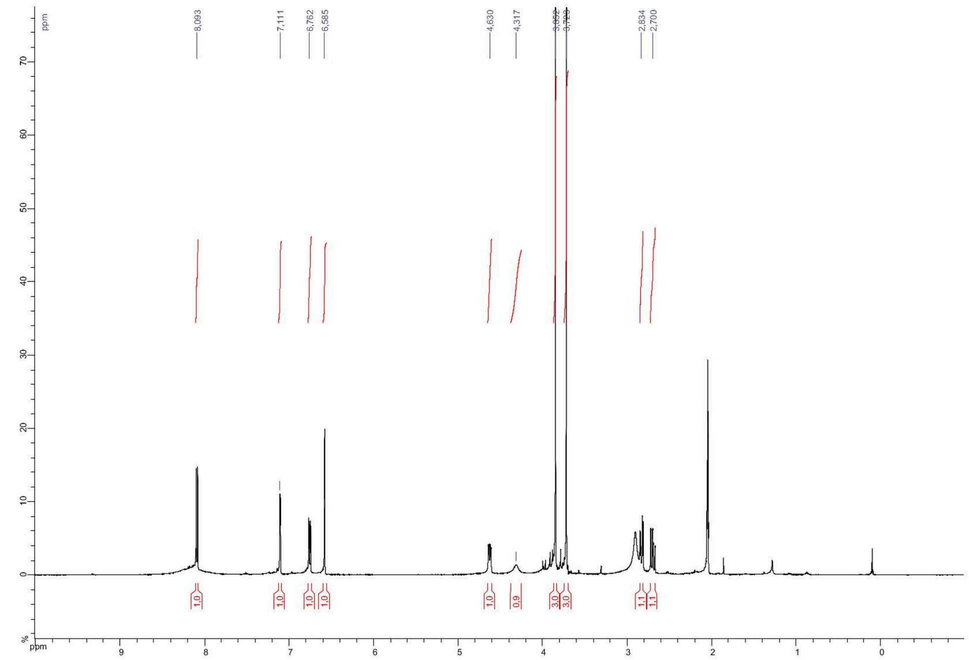

Figure S7. ^{1}H-NMR (500 MHz, acetone-d_6) spectrum of 9*S*-hydroxyerianthridin (**2**).

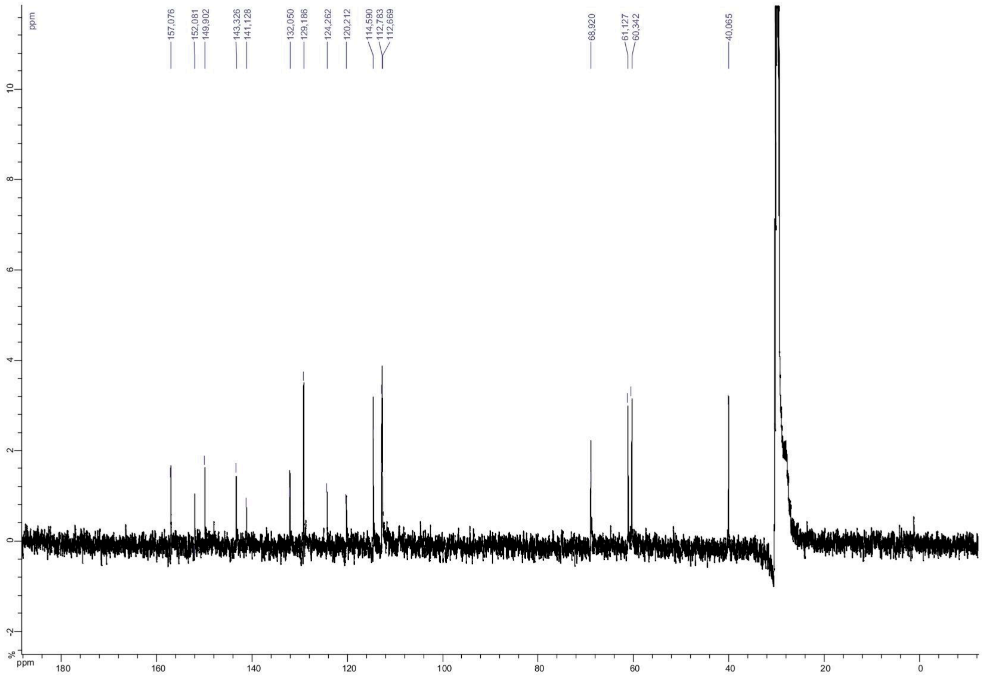

Figure S8. ^{13}C-NMR (125 MHz, acetone-d_6) spectrum of 9*S*-hydroxyerianthridin (**2**).

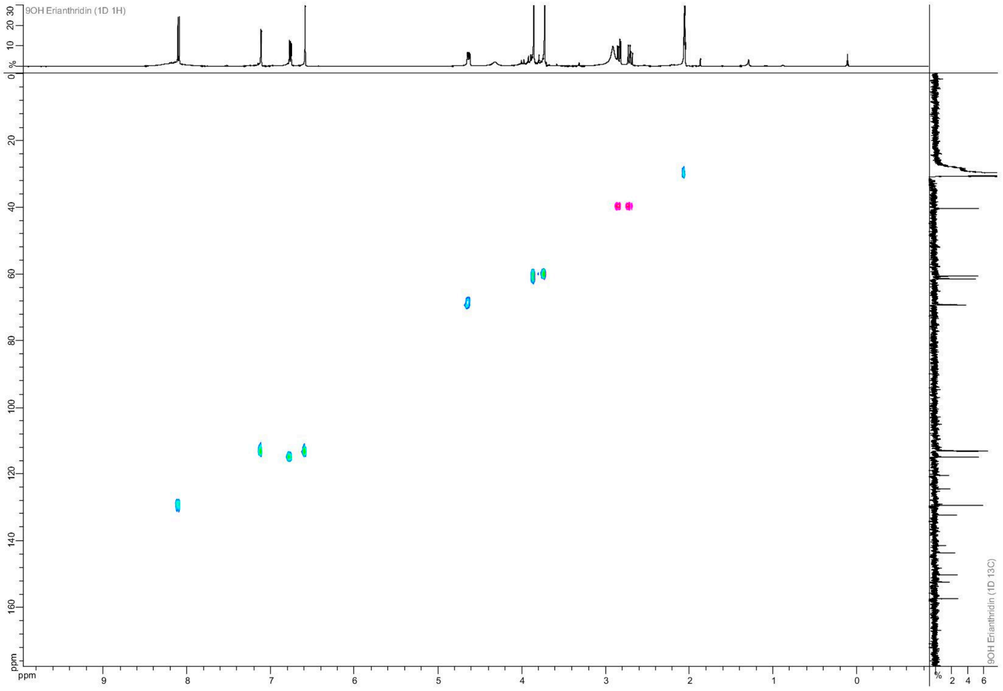

Figure S9. HSQC spectrum of 9*S*-hydroxyerianthridin (**2**).

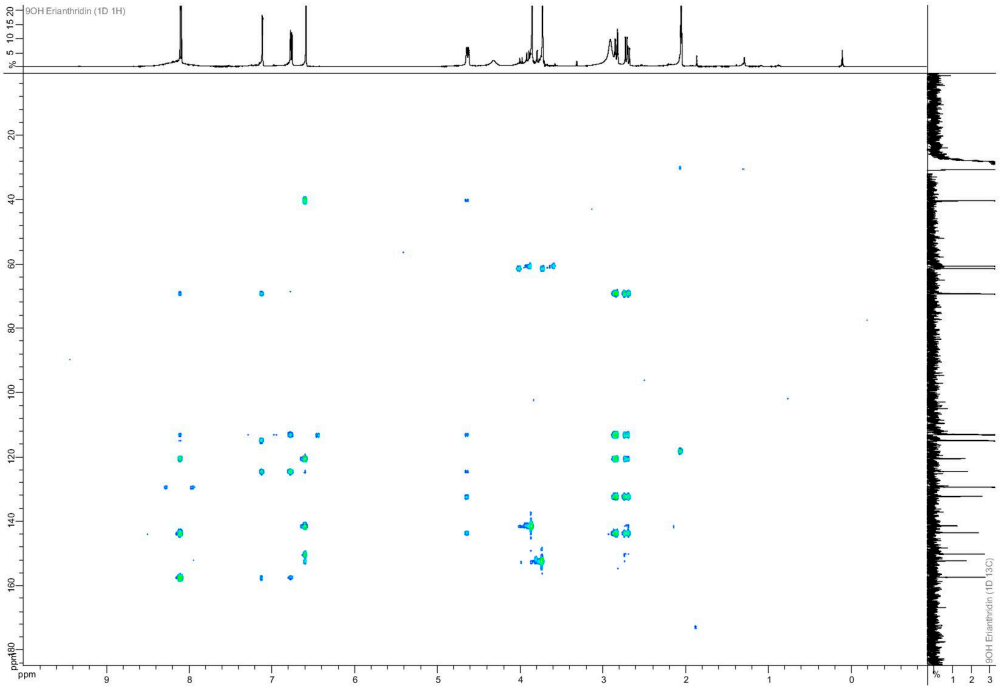

Figure S10. HMBC spectrum of 9*S*-hydroxyerianthridin (**2**).

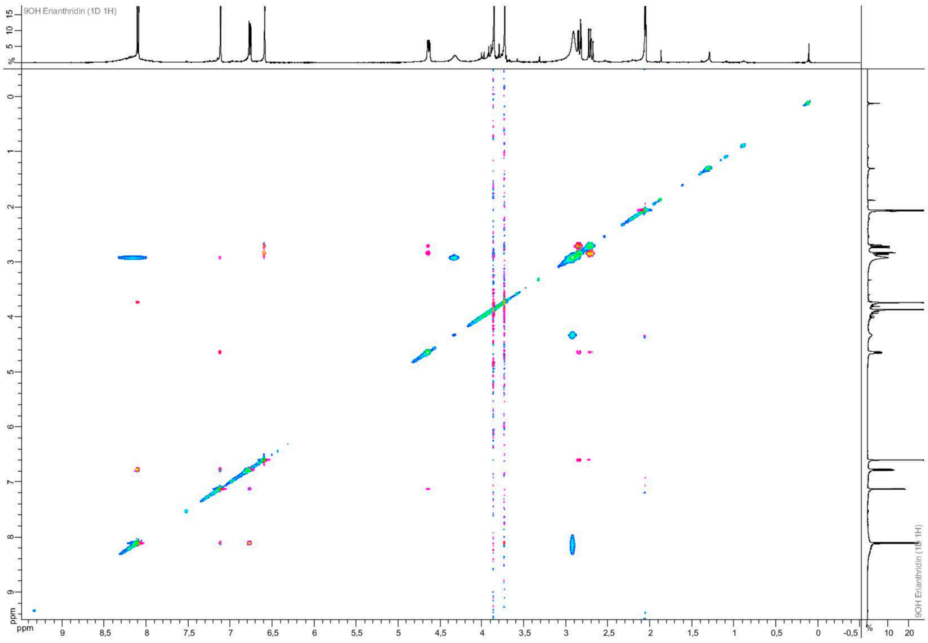

Figure S11. NOESY spectrum of 9*S*-hydroxyerianthridin (**2**).

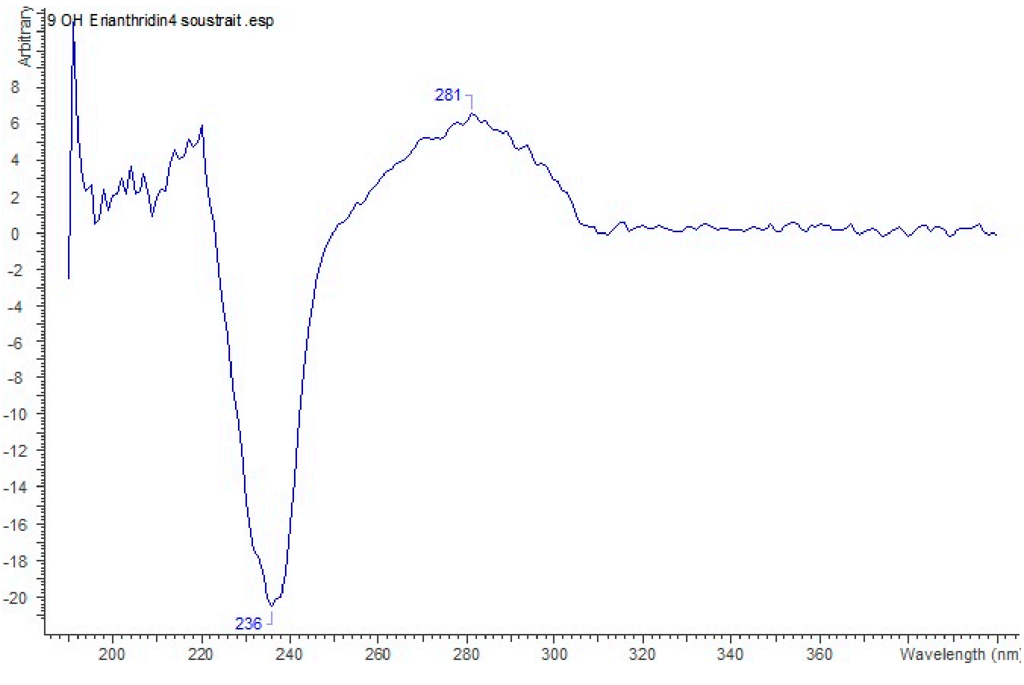

Figure S12. CD spectrum of 9*S*-Hydroxyerianthridin (**2**).

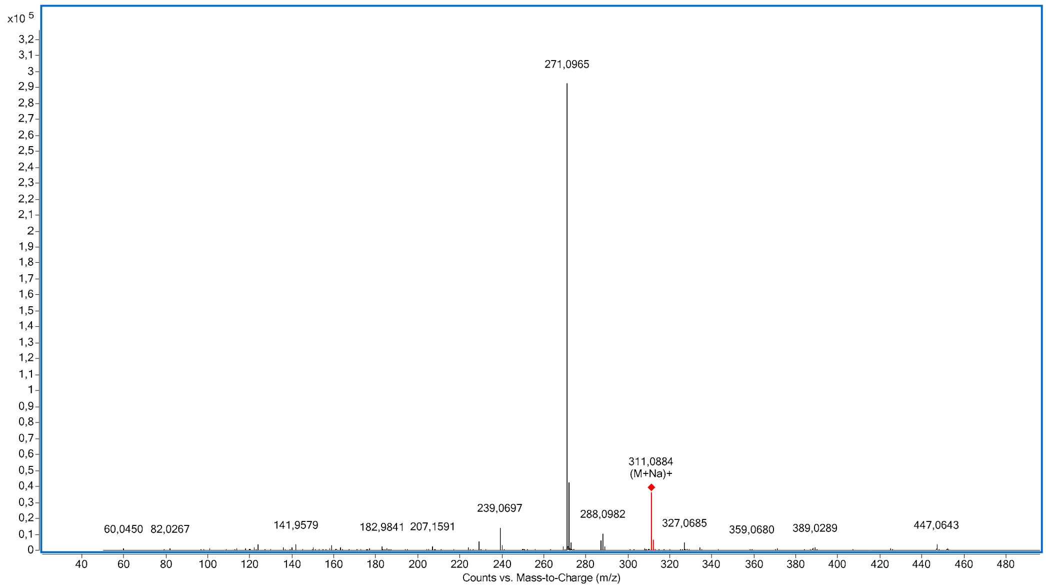

Figure S13. HRESIMS spectrum of 9*S*-Hydroxyerianthridin (**2**).

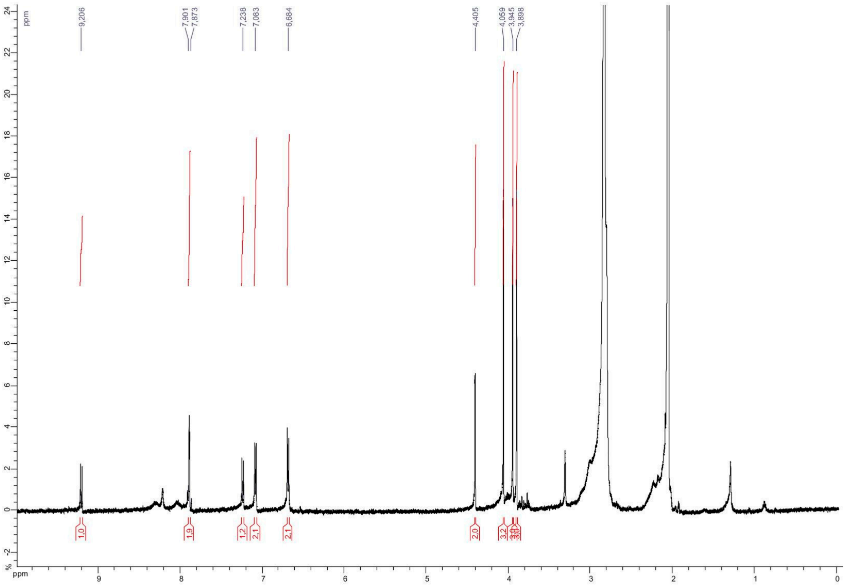

Figure S14. ^{1}H-NMR (500 MHz, acetone-d_6) spectrum of gastrodiconfusarin (**3**).

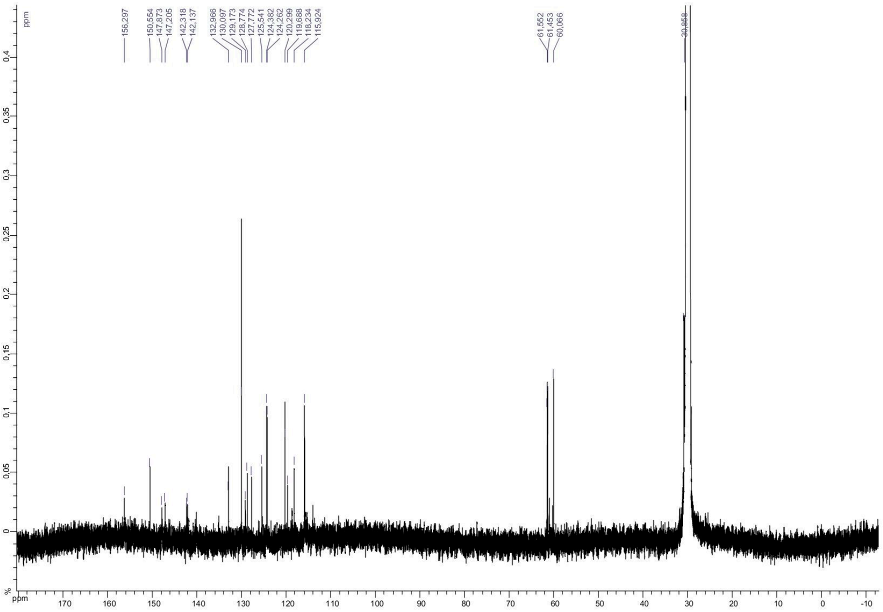

Figure S15. ^{13}C-NMR (125 MHz, acetone-d_6) spectrum of gastrodiconfusarin (**3**).

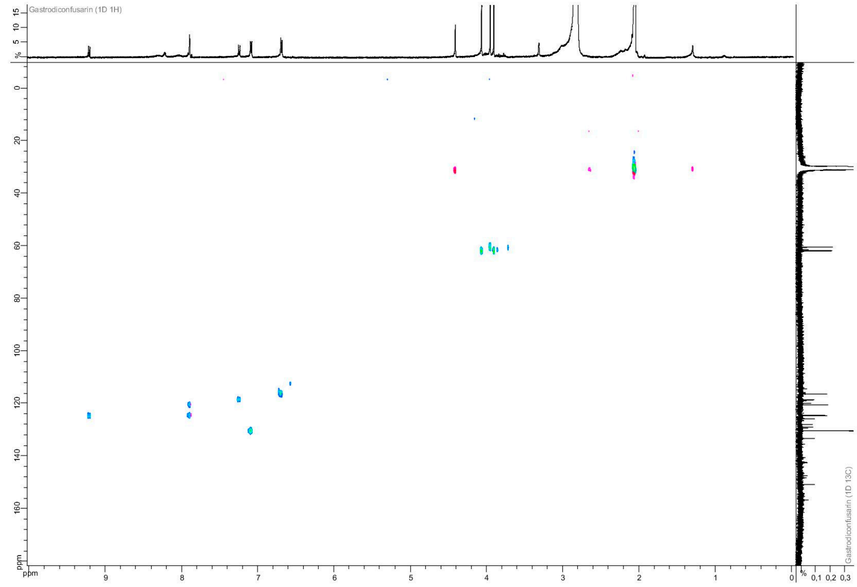

Figure S16. HSQC spectrum of gastrodiconfusarin (**3**).

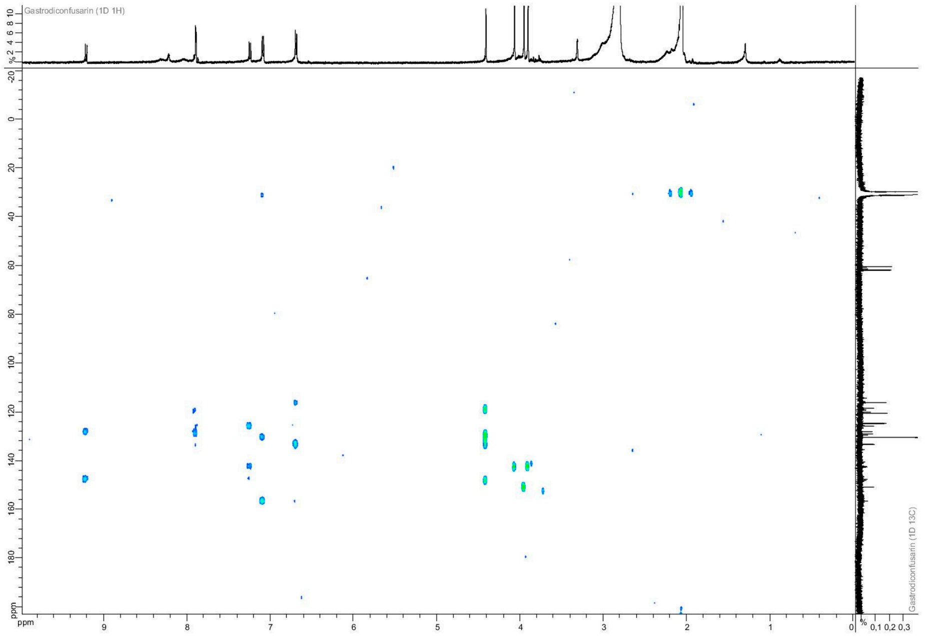

Figure S17. HMBC spectrum of gastrodiconfusarin (**3**).

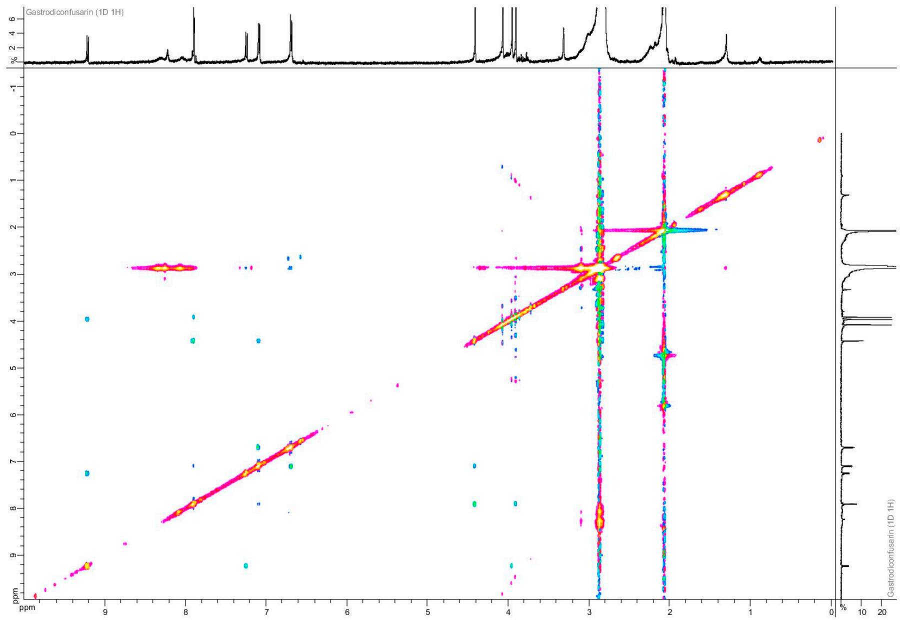

Figure S18. NOESY spectrum of gastrodiconfusarin (**3**).

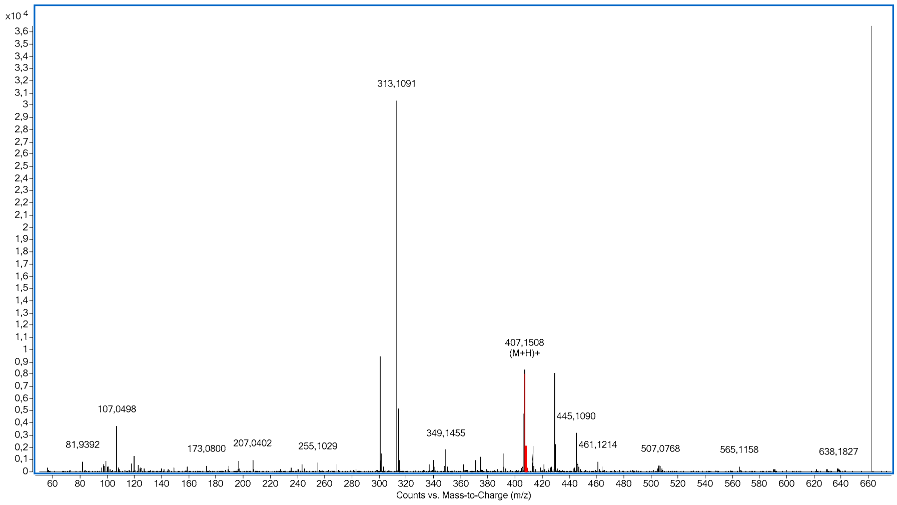

Figure S19. HRESIMS spectrum of gastrodiconfusarin (**3**).

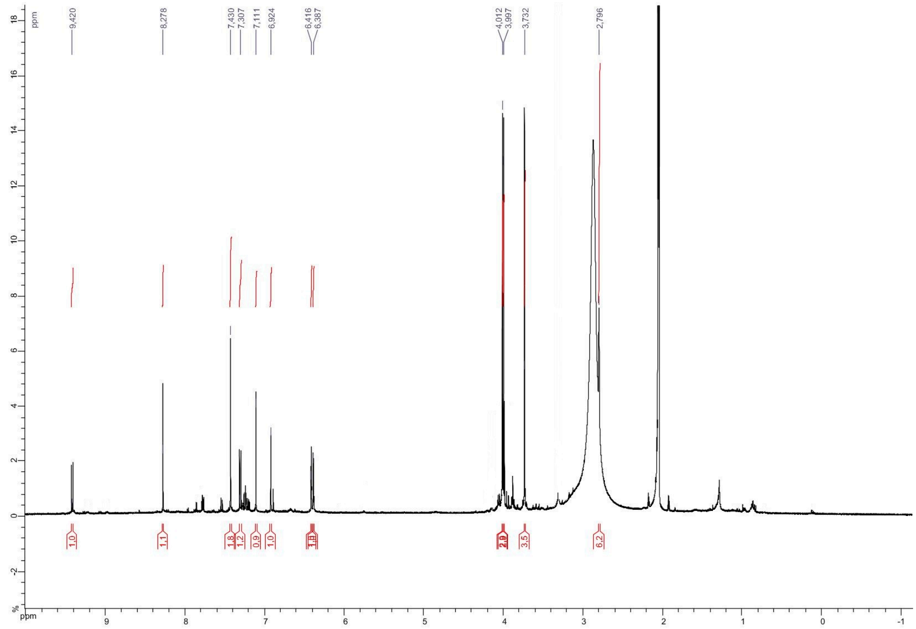

Figure S20. ^{1}H-NMR (500 MHz, acetone-d_6) spectrum of lusidol A (**4**).

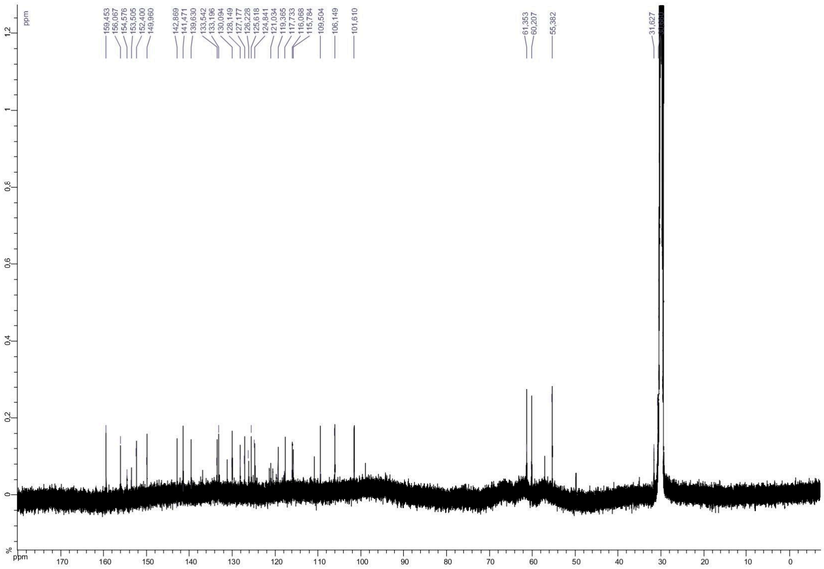

Figure S21. ^{13}C-NMR (125 MHz, acetone-d_6) spectrum of lusidol A (**4**).

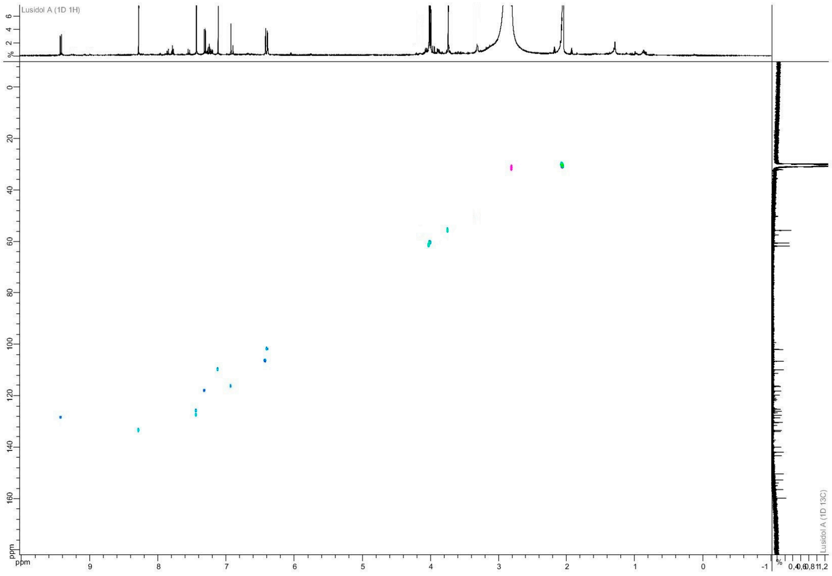

Figure S22. HSQC spectrum of lusidol A (**4**).

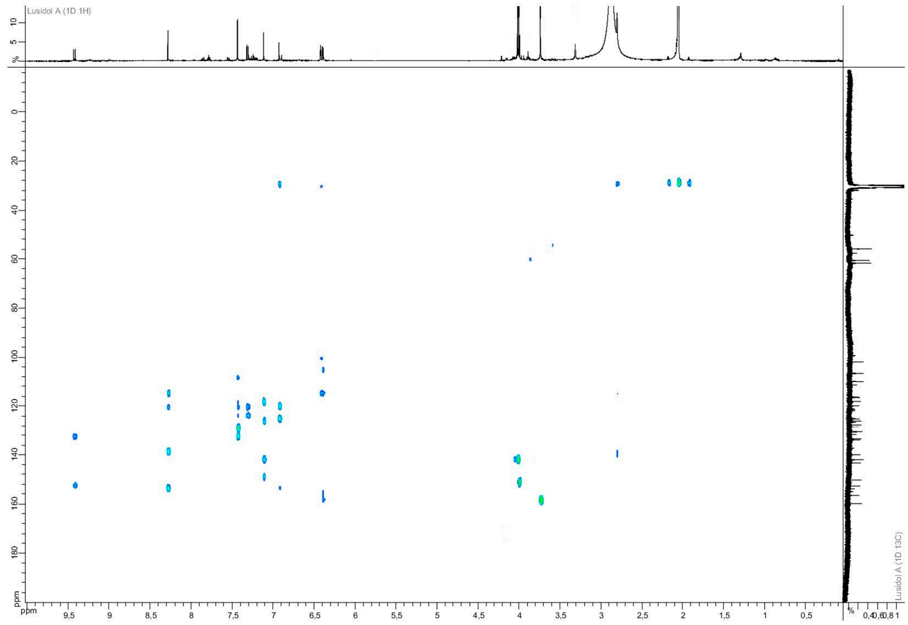

Figure S23. HMBC spectrum of lusidol A (**4**).

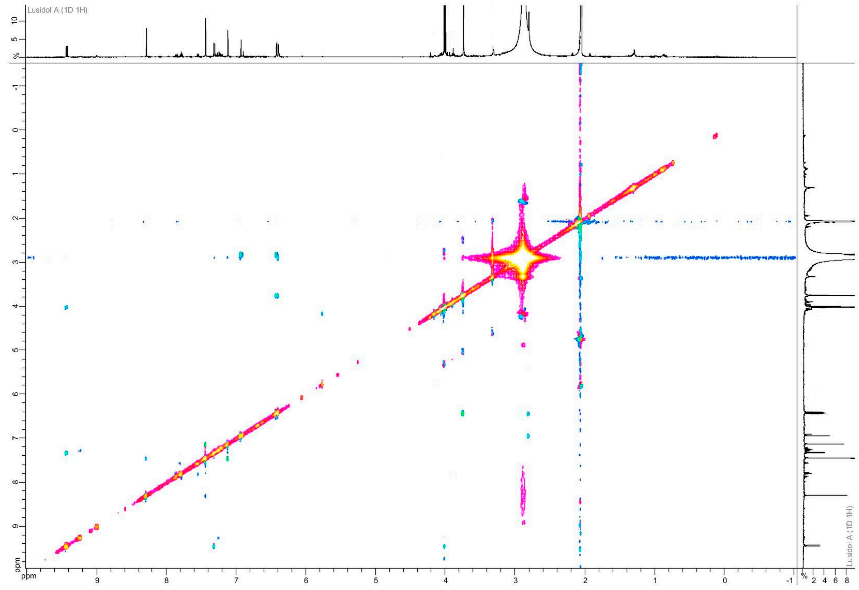

Figure S24. NOESY spectrum of lusidol A (**4**).

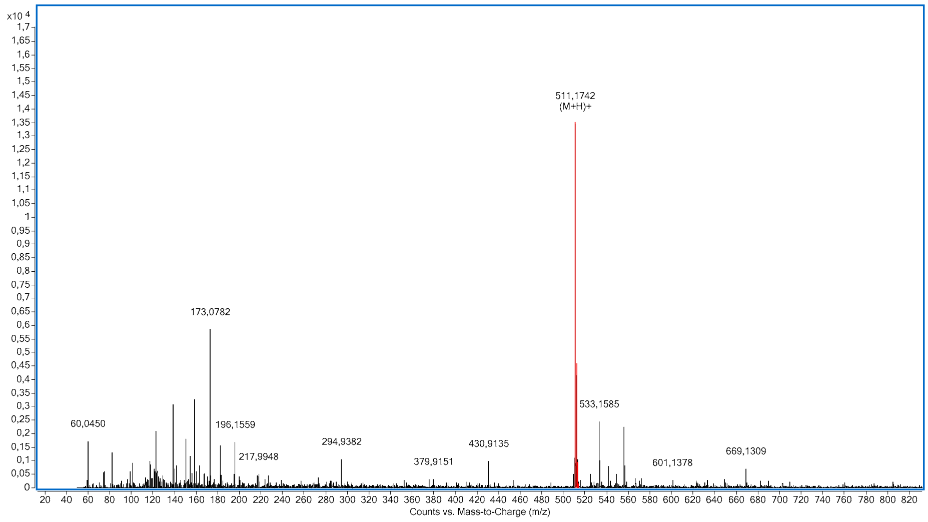

Figure S25. HRESIMS spectrum of lusidol A (**4**).

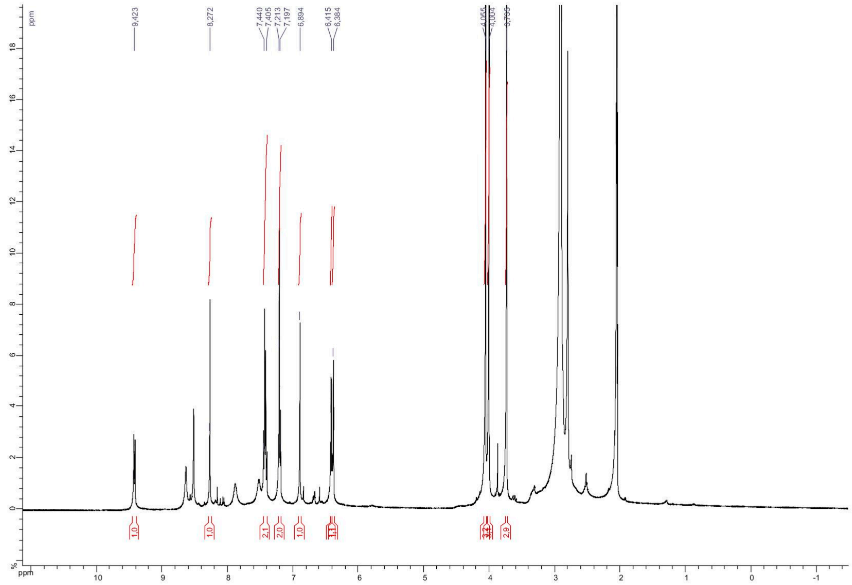

Figure S26. ^{1}H-NMR (500 MHz, acetone-d_6) spectrum of lusidol B (**5**).

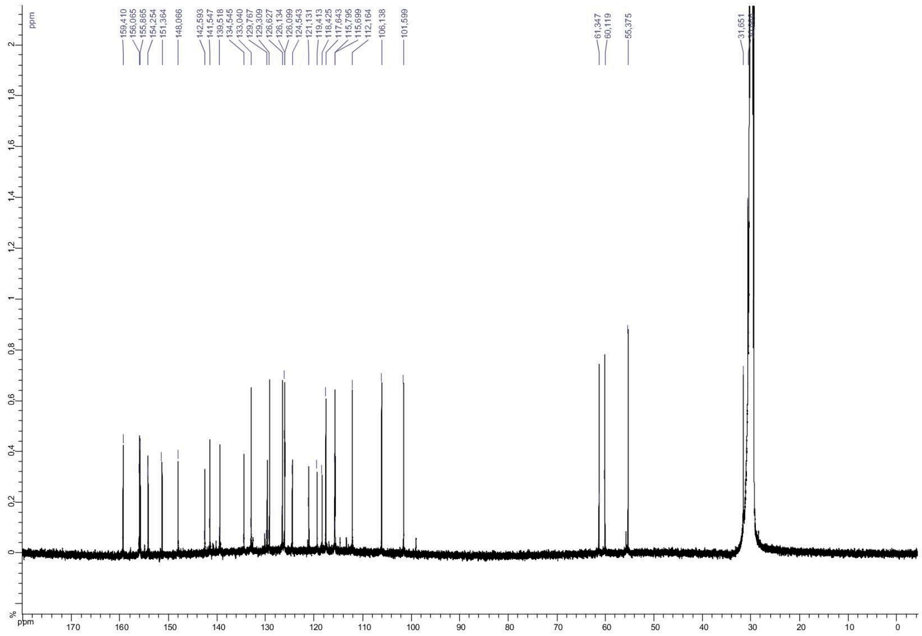

Figure S27. ^{13}C-NMR (125 MHz, acetone-d_6) spectrum of lusidol B (**5**).

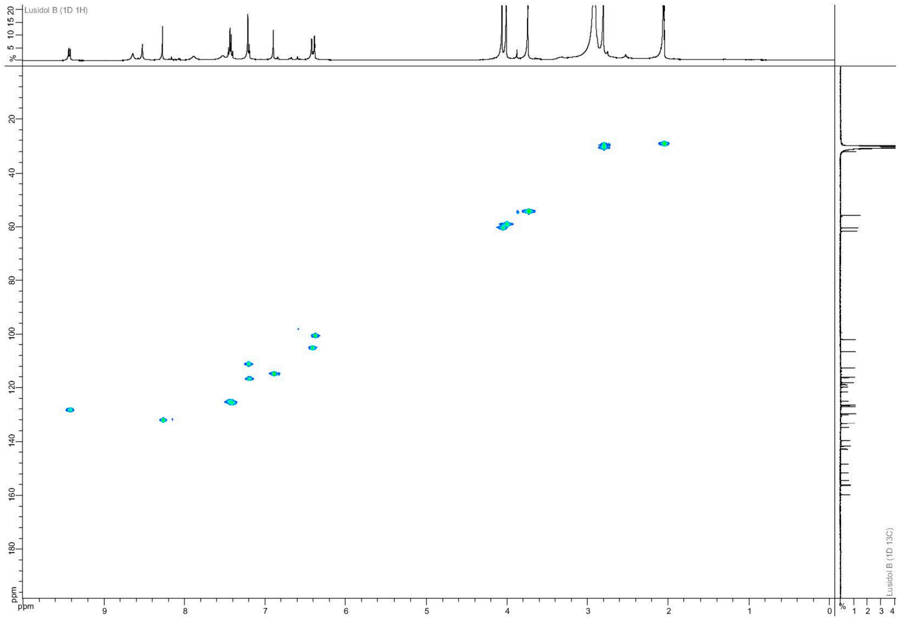

Figure S28. HSQC spectrum of lusidol B (**5**).

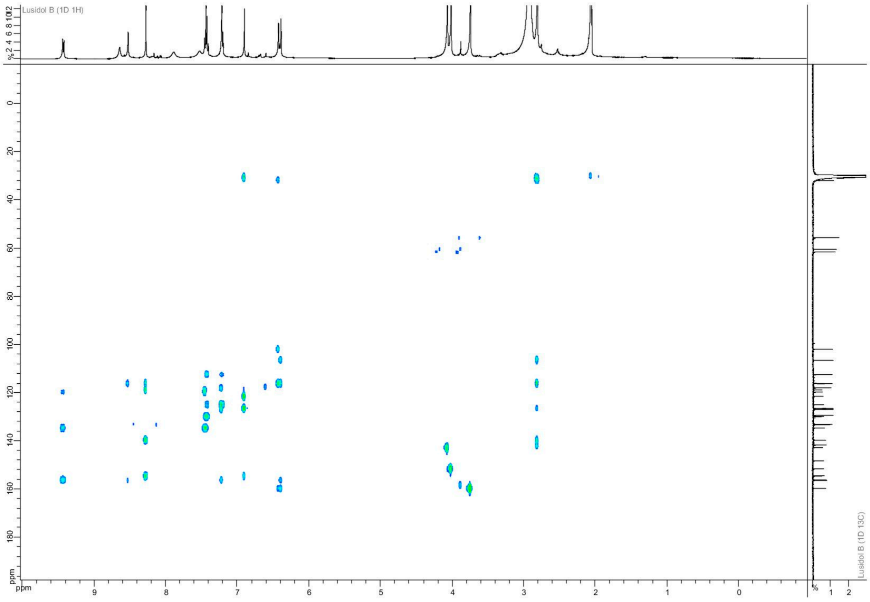

Figure S29. HMBC spectrum of lusidol B (**5**).

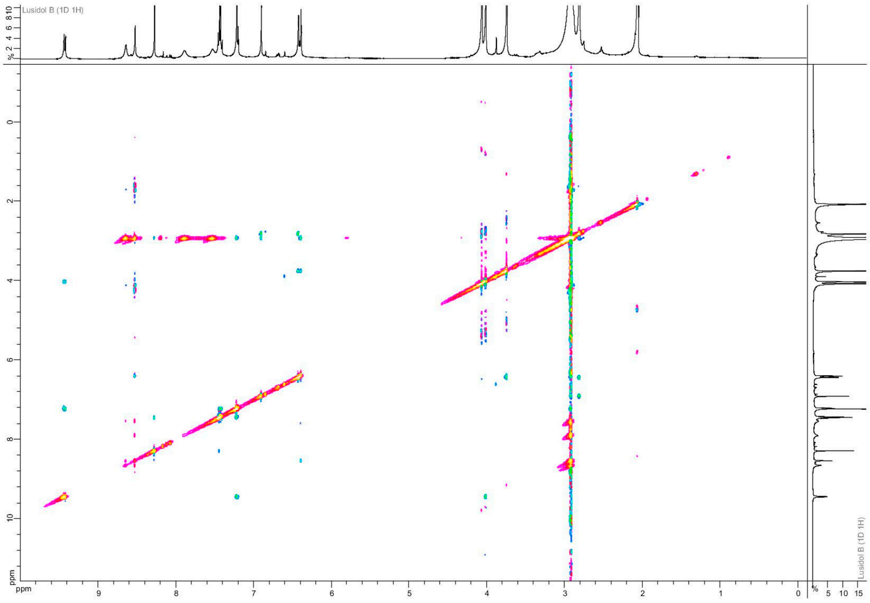

Figure S30. NOESY spectrum of lusidol B (**5**).

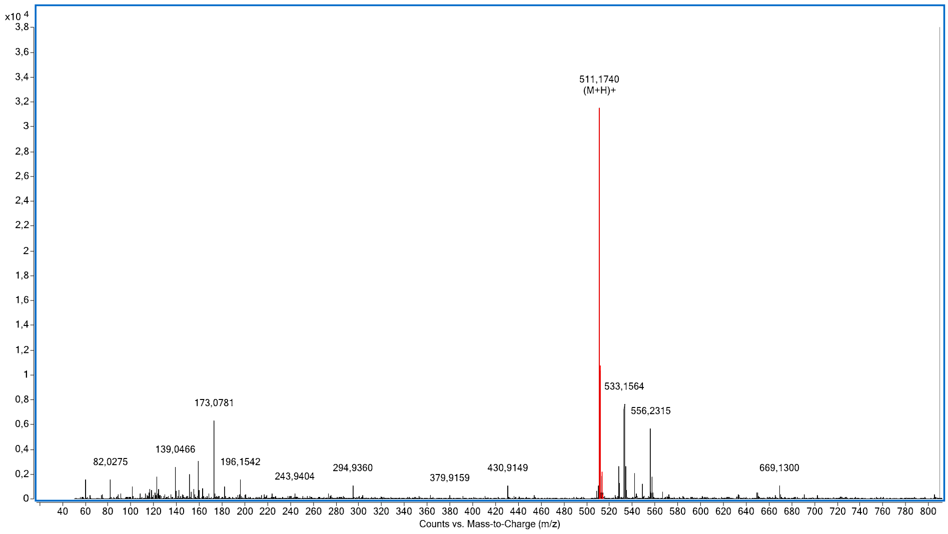

Figure S31. HRESIMS spectrum of lusidol B (**5**).

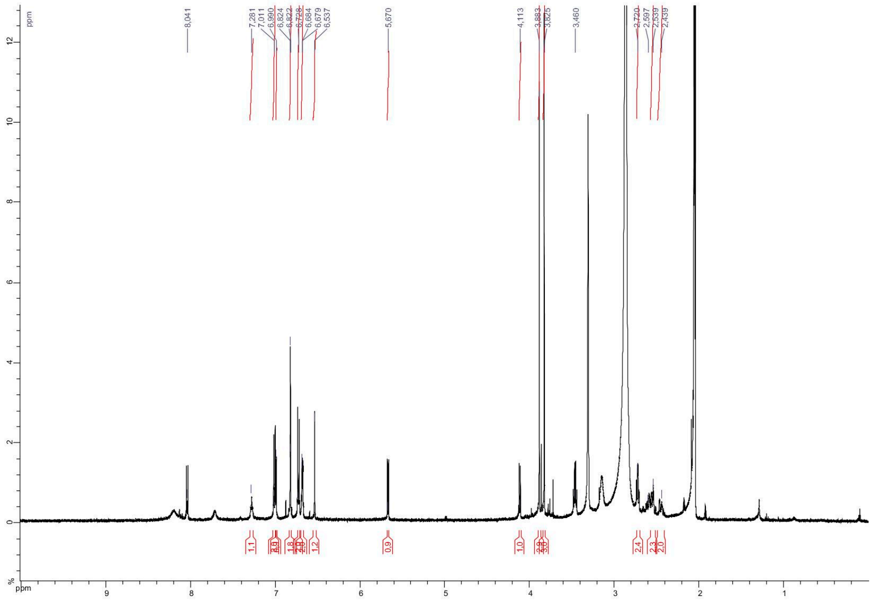

Figure S32. ^{1}H-NMR (500 MHz, acetone-d_6) spectrum of moupilonin (**6**).

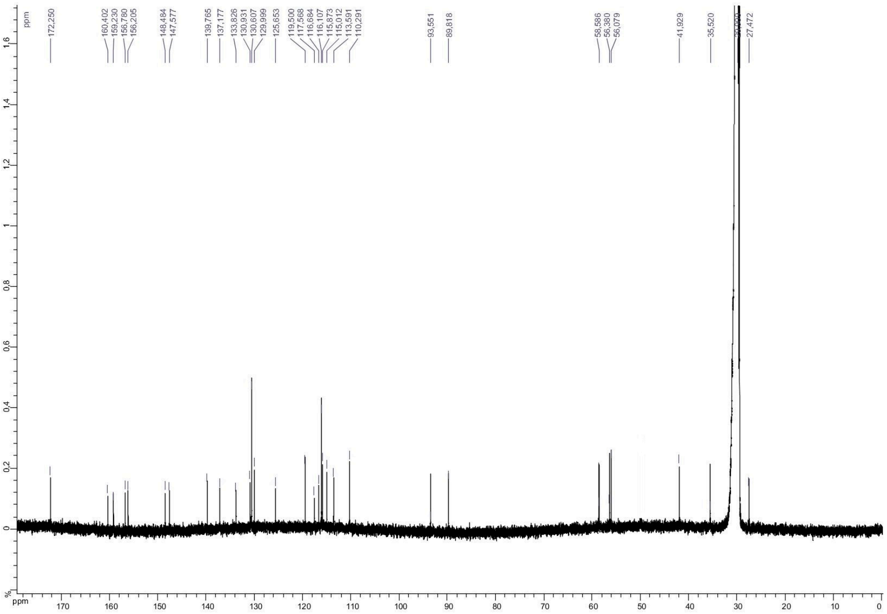

Figure S33. ^{13}C-NMR (125 MHz, acetone-d_6) spectrum of moupilonin (**6**).

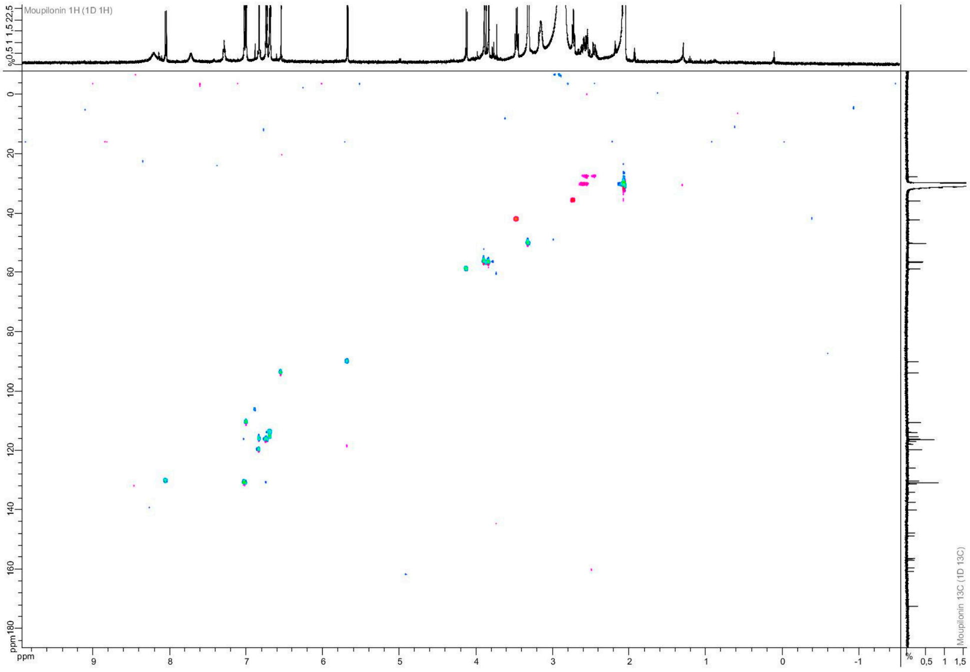

Figure S34. HSQC spectrum of moupilonin (**6**).

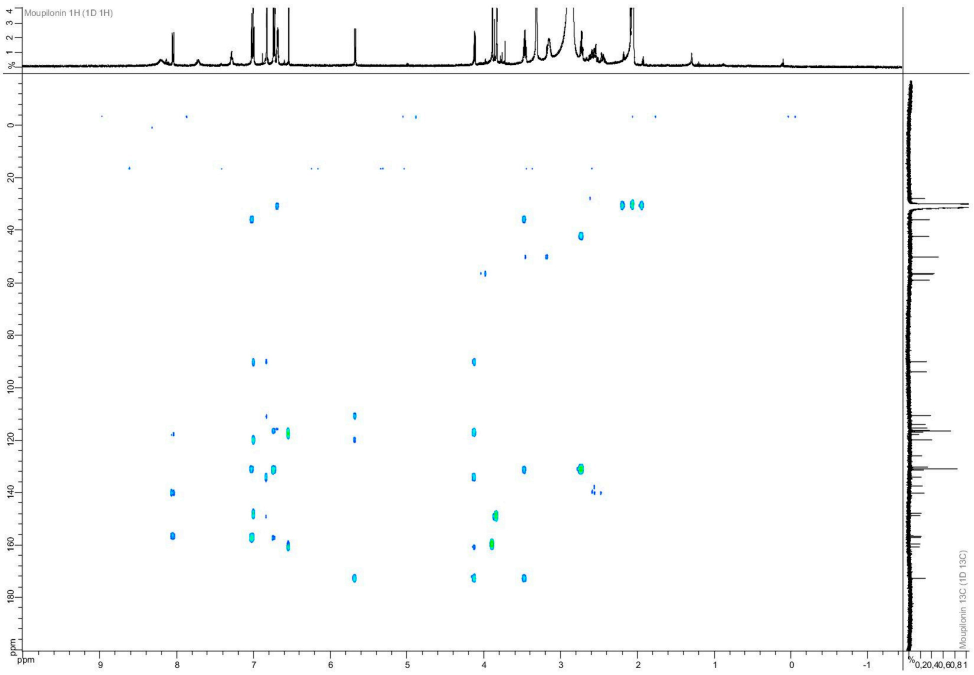

Figure S35. HMBC spectrum of moupilonin (**6**).

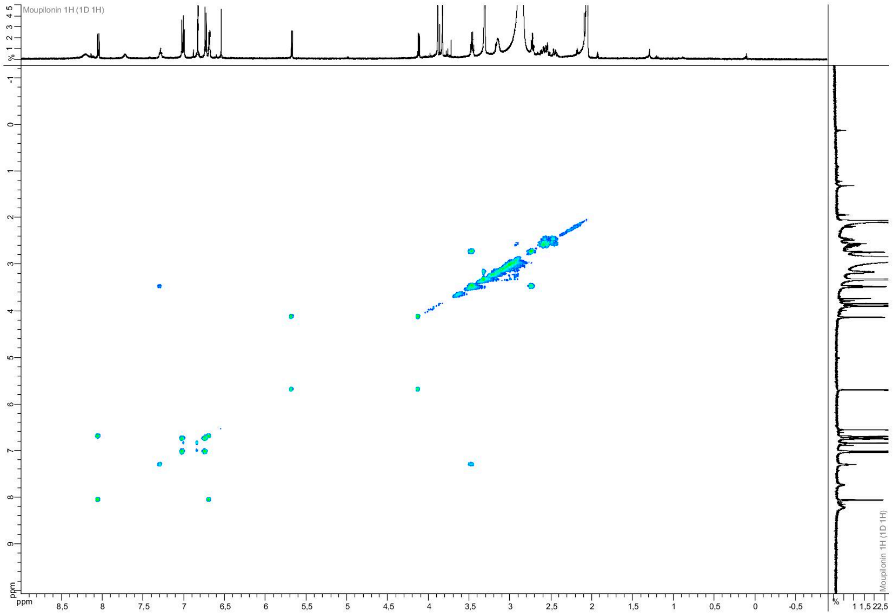

Figure S36. COSY spectrum of moupilonin (**6**).

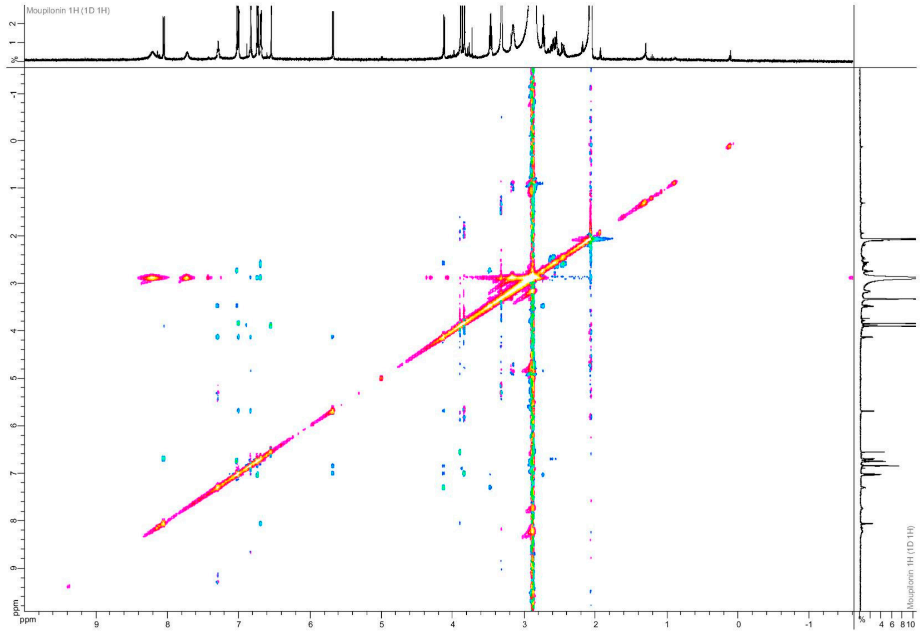

Figure S37. NOESY spectrum of moupilonin (**6**).

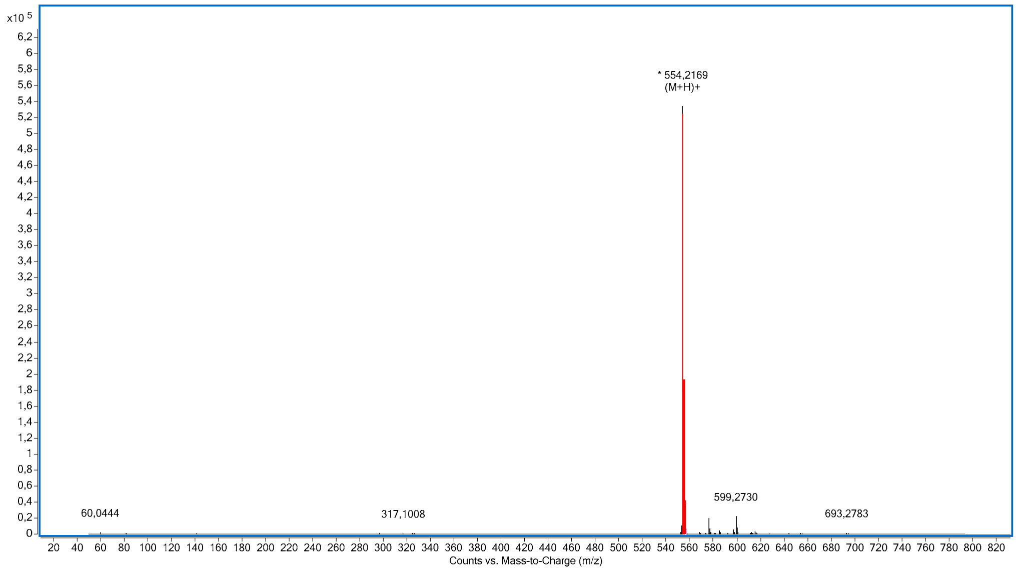

Figure S38. HRESIMS spectrum of moupilonin (**6**).

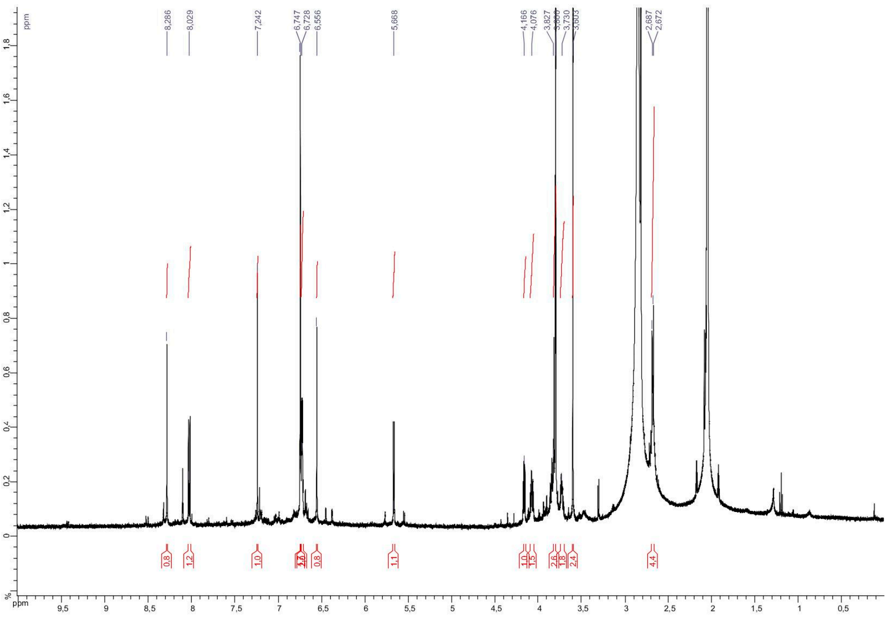

Figure S39. ^{1}H-NMR (500 MHz, acetone-d_6) spectrum of cyrtonesin A (**7**).

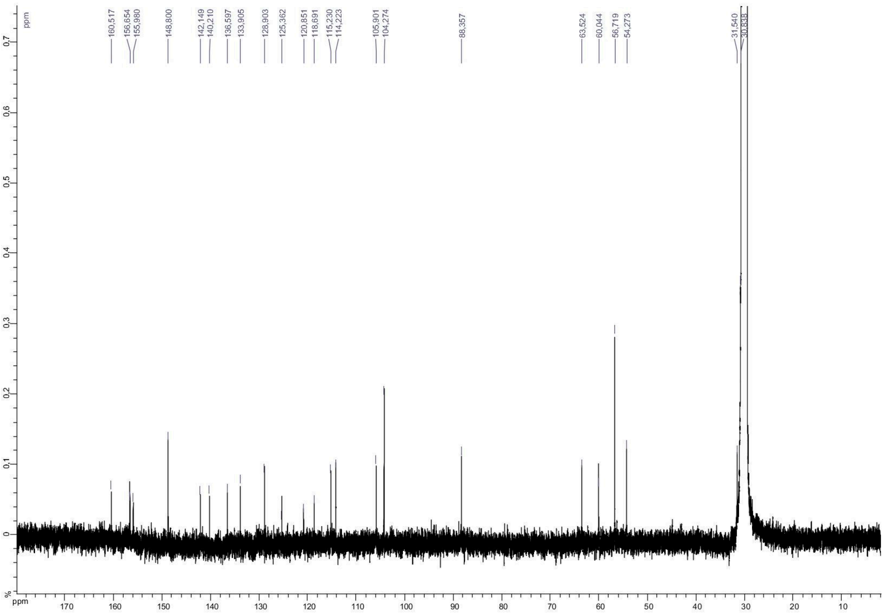

Figure S40. ^{13}C-NMR (125 MHz, acetone-d_6) spectrum of cyrtonesin A (**7**).

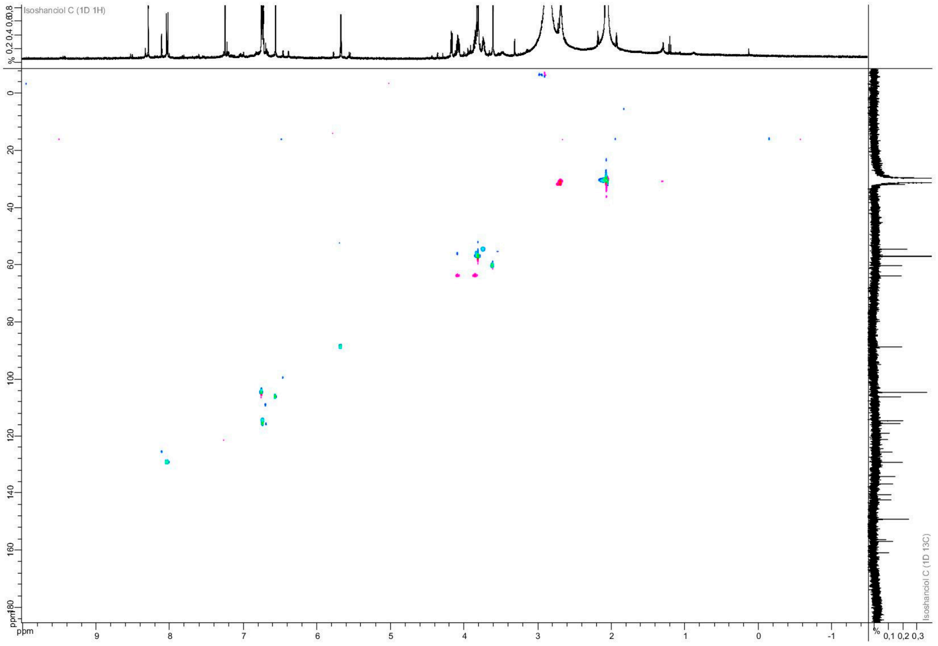

Figure S41. HSQC spectrum of cyrtonesin A (**7**).

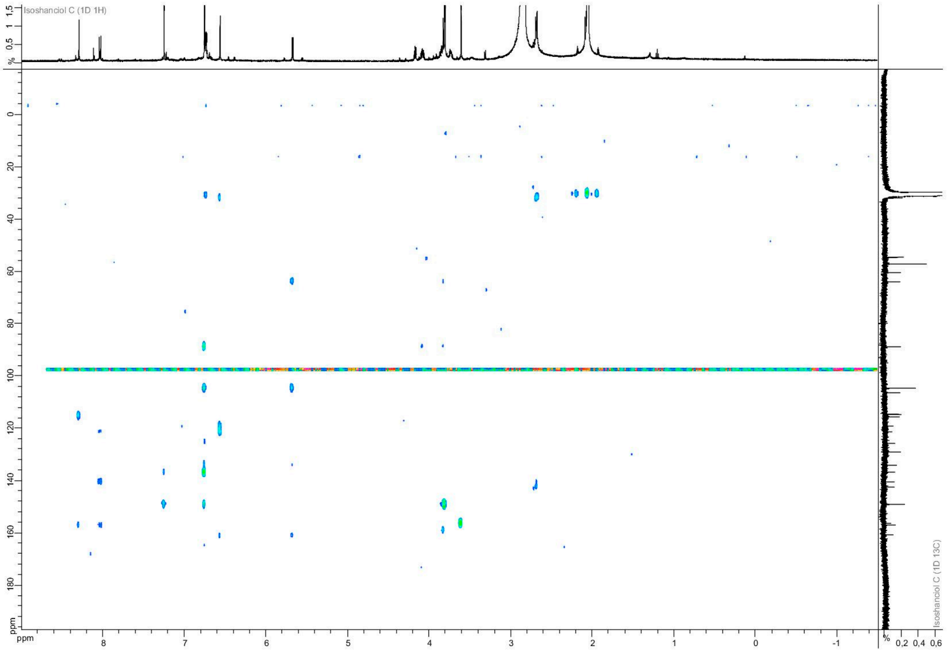

Figure S42. HMBC spectrum of cyrtonesin A (**7**).

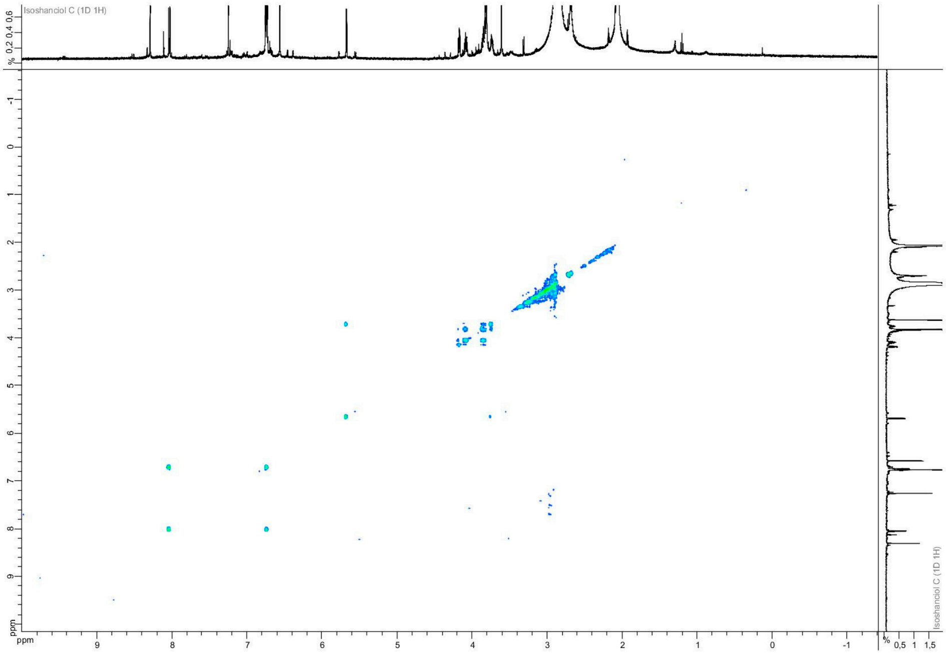

Figure S43. COSY spectrum of cyrtonesin A (**7**).

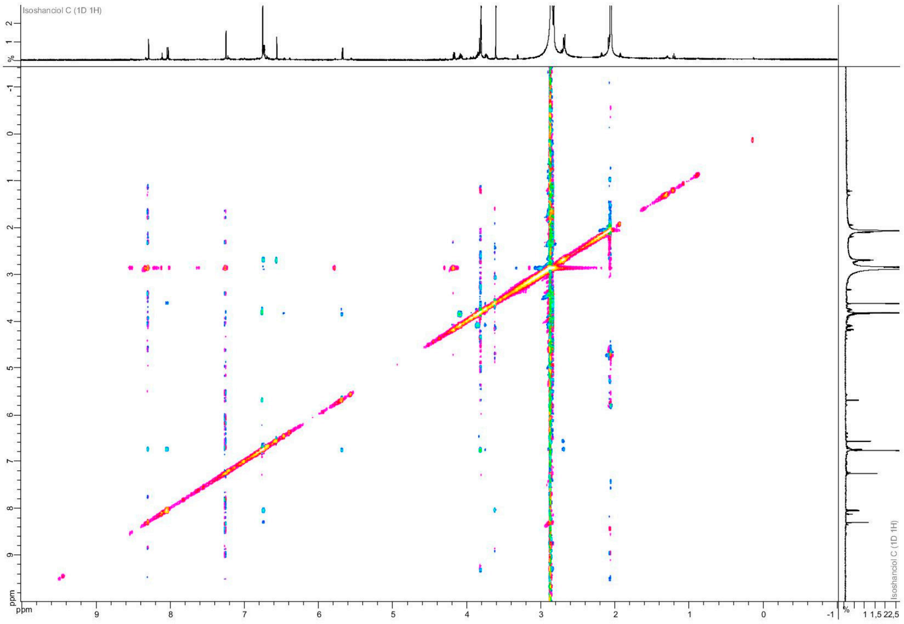

Figure S44. NOESY spectrum of cyrtonesin A (**7**).

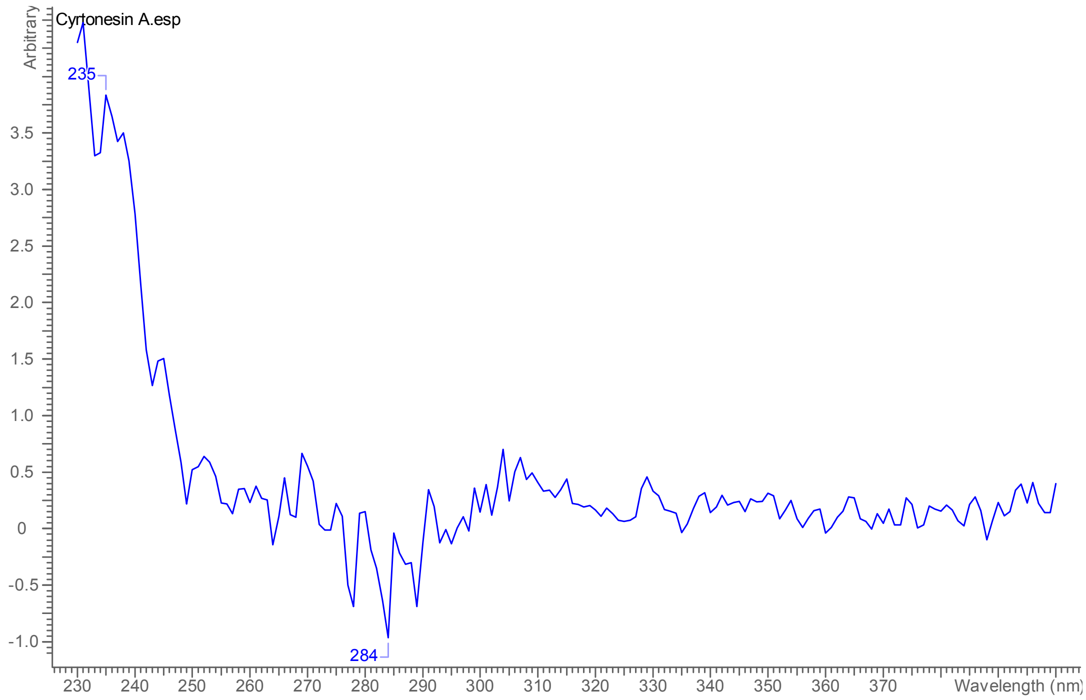

Figure S45. CD spectrum of cyrtonesin A (**7**).

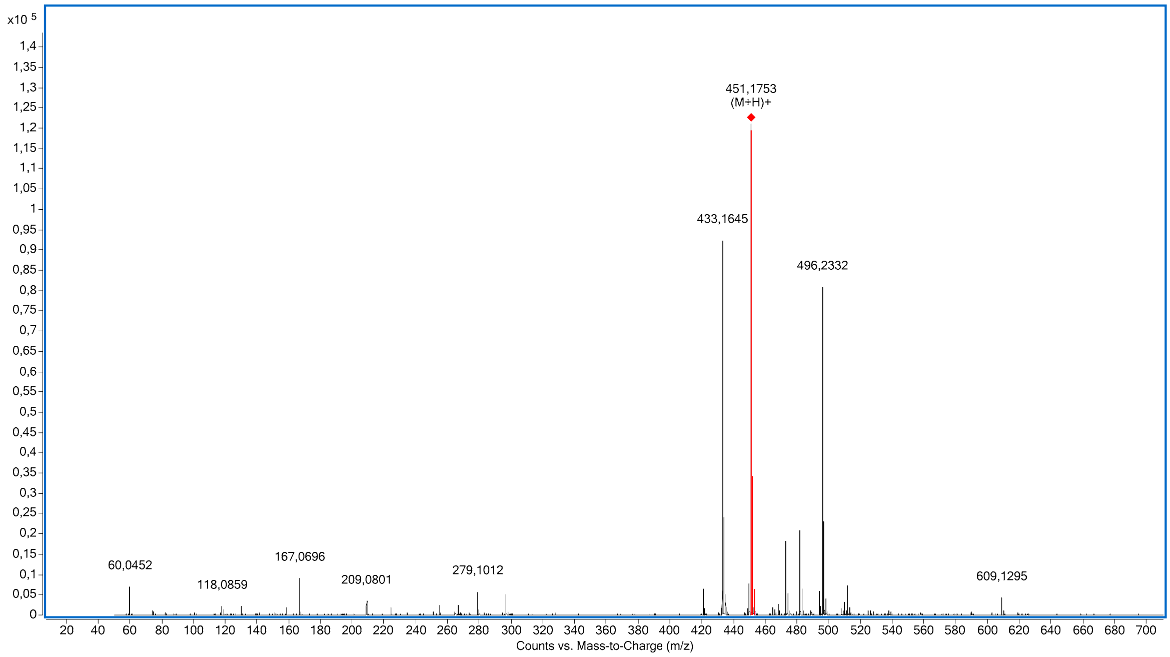

Figure S46. HRESIMS spectrum of cyrtonesin A (**7**).

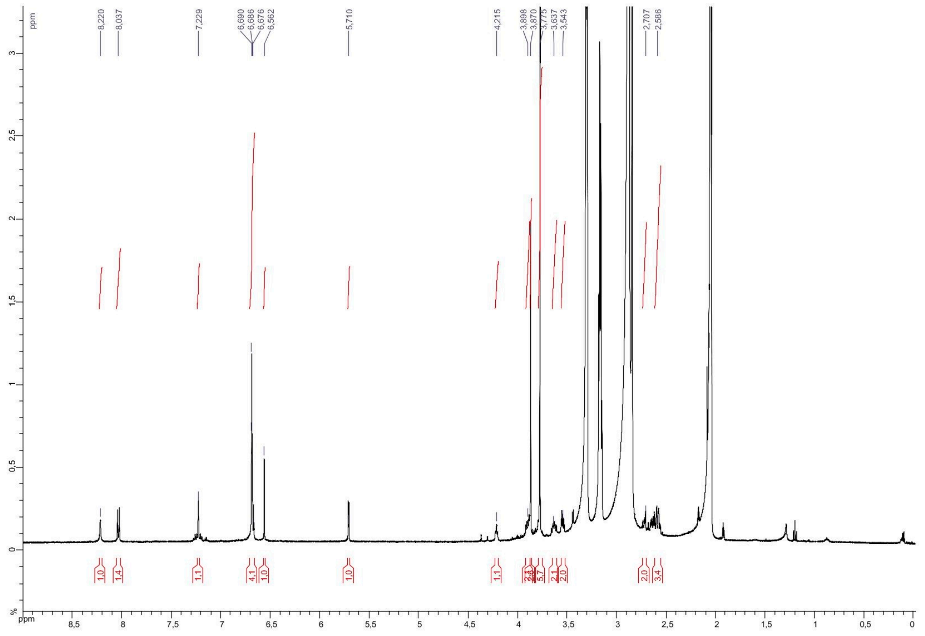

Figure S47. ^{1}H-NMR (500 MHz, acetone-d_6) spectrum of cyrtonesin B (**8**).

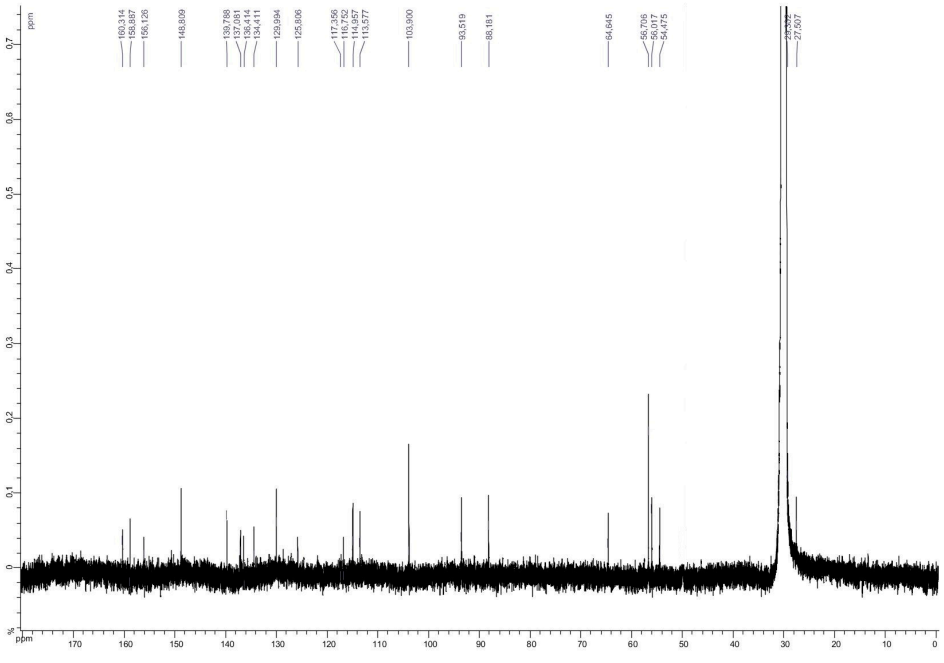

Figure S48. ^{13}C-NMR (500 MHz, acetone-d_6) spectrum of cyrtonesin B (**8**).

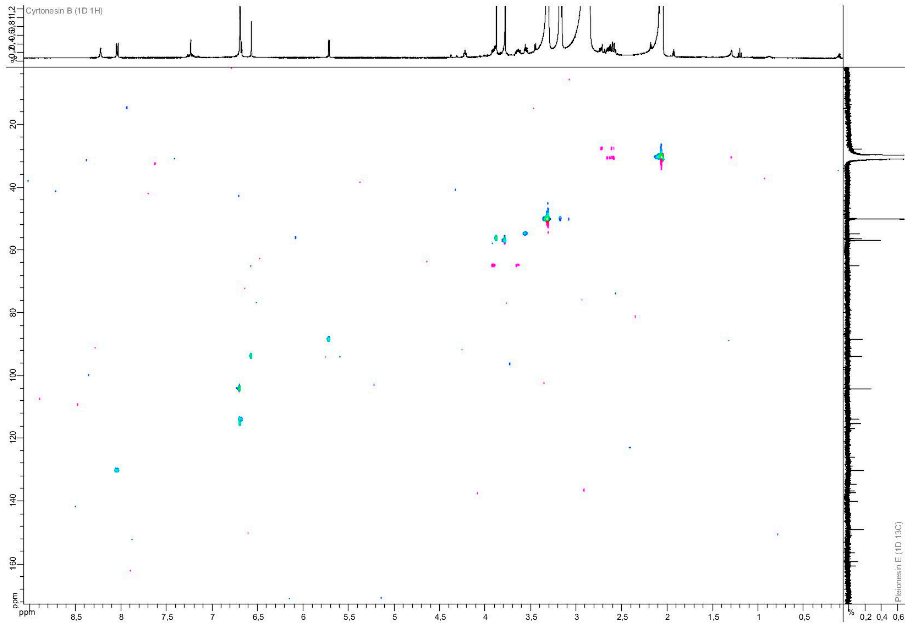

Figure S49. HSQC spectrum of cyrtonesin B (**8**).

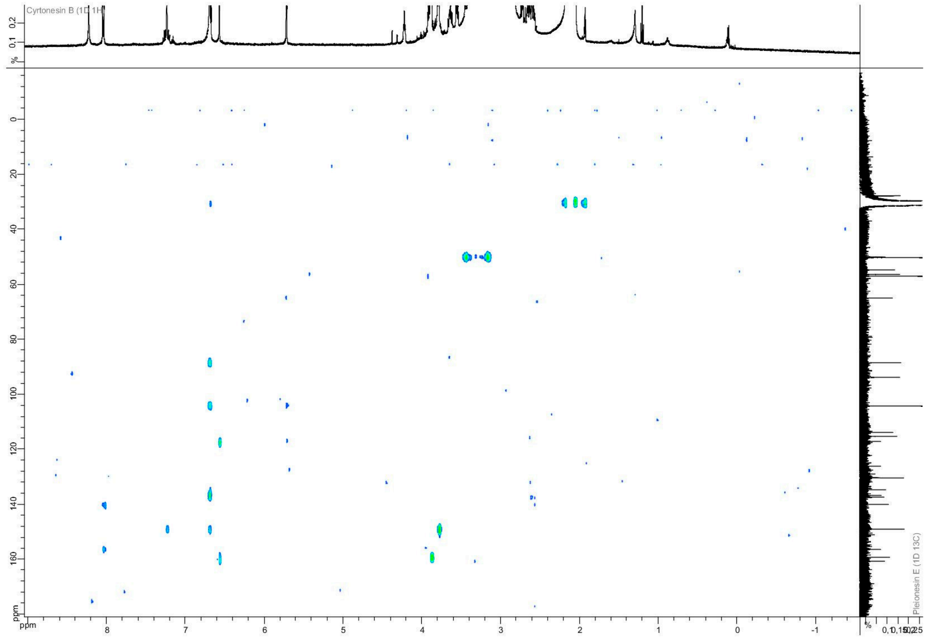

Figure S50. HMBC spectrum of cyrtonesin B (**8**).

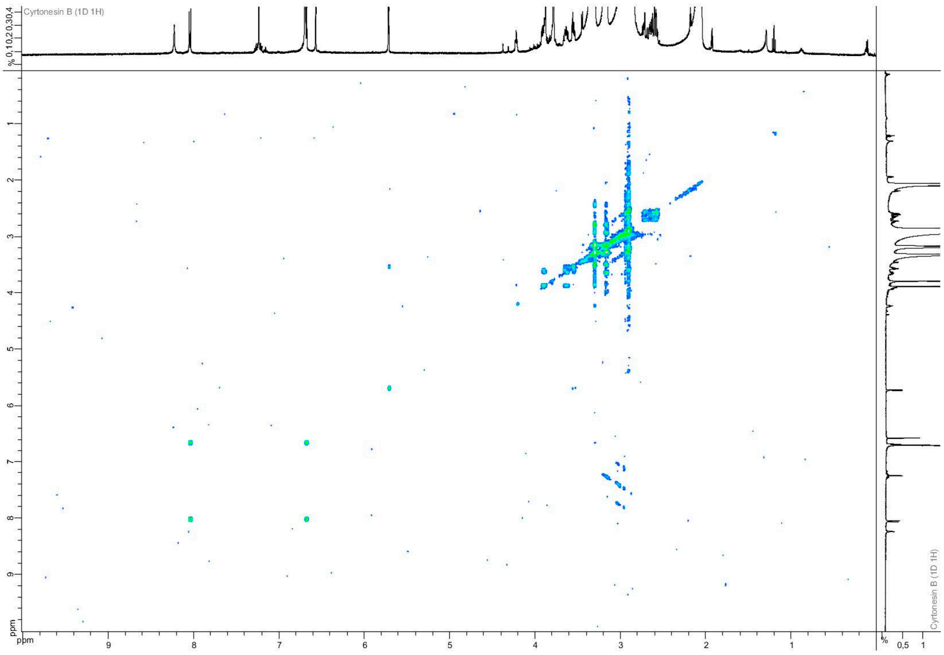

Figure S51. COSY spectrum of cyrtonesin B (**8**).

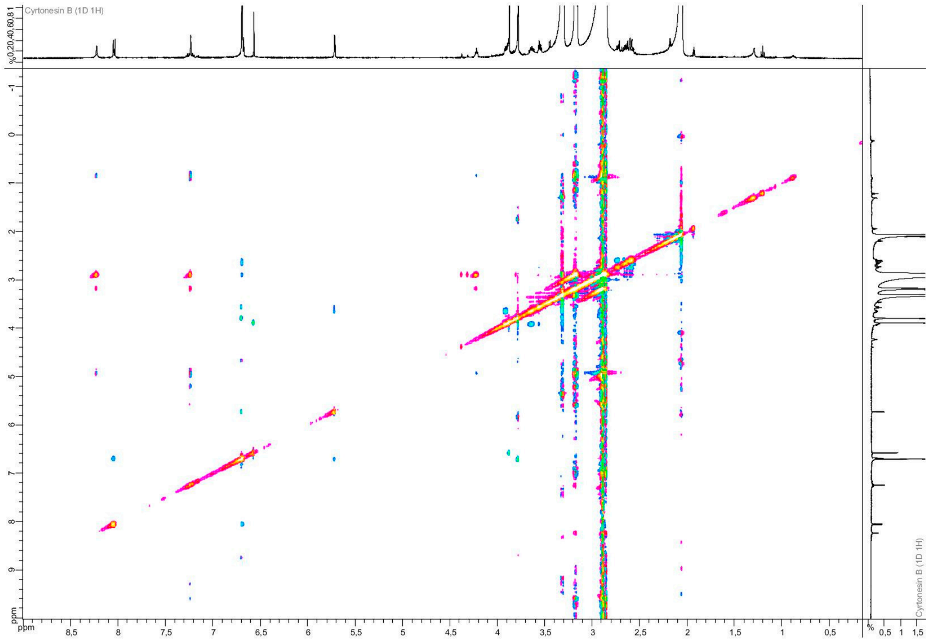

Figure S52. NOESY spectrum of cyrtonesin B (**8**).

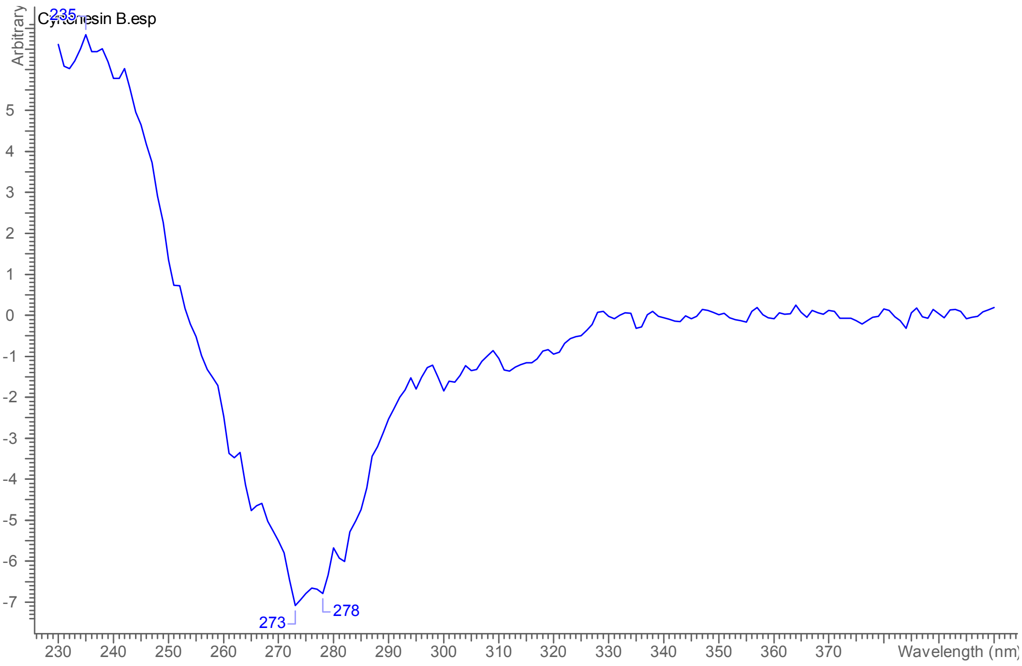

Figure S53. CD spectrum of cyrtonesin B (**8**).

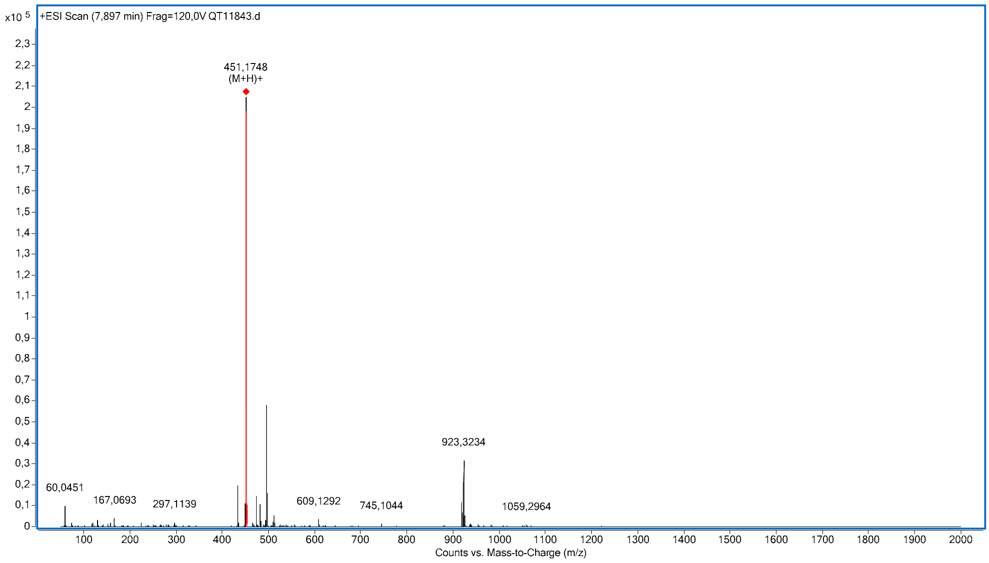

Figure S54. HRESIMS spectrum of cyrtonesin B (**8**).
